# Nanobiosystems for Antimicrobial Drug-Resistant Infections

**DOI:** 10.3390/nano11051075

**Published:** 2021-04-22

**Authors:** Foteini Gkartziou, Nikolaos Giormezis, Iris Spiliopoulou, Sophia G. Antimisiaris

**Affiliations:** 1 Institute of Chemical Engineering, FORTH/ICES, Platani, 26504 Patras, Greece; fotini_gartz@yahoo.gr; 2 National Reference Centre for Staphylococci, School of Medicine, University of Patras, 26504 Patras, Greece; giormenik@yahoo.gr; 3 Department of Microbiology, School of Medicine, University of Patras, 26504 Patras, Greece; 4 Laboratory of Pharmaceutical Technology, Department of Pharmacy, University of Patras, 26504 Patras, Greece

**Keywords:** antimicrobial resistance, MRSA, nanoparticle, liposomes, drug, bacteriophage, infectious disease

## Abstract

The worldwide increased bacterial resistance toward antimicrobial therapeutics has led investigators to search for new therapeutic options. Some of the options currently exploited to treat drug-resistant infections include drug-associated nanosystems. Additionally, the use of bacteriophages alone or in combination with drugs has been recently revisited; some studies utilizing nanosystems for bacteriophage delivery have been already reported. In this review article, we focus on nine pathogens that are the leading antimicrobial drug-resistant organisms, causing difficult-to-treat infections. For each organism, the bacteriophages and nanosystems developed or used in the last 20 years as potential treatments of pathogen-related infections are discussed. Summarizing conclusions and future perspectives related with the potential of such nano-antimicrobials for the treatment of persistent infections are finally highlighted.

## 1. Introduction

This paper is a systematic review of the recent efforts (last 20 years) to apply nan-otechnological approaches in order to improve the delivery of antimicrobial drugs (or drug combinations), for improved treatment of resistant microbial diseases. The review is focused on the leading antimicrobial drug-resistant organisms: (i) Methicillin-resistant *Staphylococcus aureus* (MRSA); (ii) Vancomycin-resistant Enterococci (VRE); (iii) *Clostridium difficile* (*C. difficile*); (iv) Carbapenem-resistant enterobacte-riaceae (CRE); (v) *Pseudomonas aeruginosa* carbapenem-resistant (PACR); (vi) Carbapenem-resistant *Acinetobacter baumannii* (CRAB); (vii) *Neisseria gonnorhoeae* (Ng); (viii) *Mycobacterium tuberculosis* (MTB); and (ix) Mycobacteria other than tu-berculosis (MOTT).

Detailed literature searches were initially performed for each pathogen, using the Pub Med database [1] and the key words: <the name of the microbe> AND (<liposome> OR <nanoparticle>) AND (<drug> or <bacteriophage>). From the total pool of the hits of each search, the hits were screened for selection of the particular papers focusing on the development of nanosystems for the delivery of antimicrobial therapeutic agents, excluding those in which metal nanoparticles (NPs) are evaluated (alone) for their antimicrobial activity; only the reports in which metal NPs are associated with drugs were included. In a second step, the hits were categorized into two groups, according to the type of nanosystem developed; the lipidic (or lipid-based) NPs and the non-lipidic NPs. Finally, the most advanced studies from each group that include at least in vitro evaluation of the antimicrobial potential of the particular nanosystem developed (if not also in vivo) were selected and are discussed below.

As a special category of antimicrobials, we include herein the recently revisited approach of antimicrobial bacteriophage therapeutics (as mentioned above), if such cases have been reported for the specific pathogen discussed. Additionally, approaches considering the encapsulation of bacteriophages in nanotechnologies, such as liposomes (LIPs), with the aim of increasing their stability and antimicrobial activity, are also included.

In the first part of this review, a brief description of the structure, components, and properties of the various types of nanosystems or nanoparticles used as drug carriers [2,3,4,5] is given. In addition, the categories and mechanisms of action of the antimicrobial therapeutics that are currently used to treat infections are briefly mentioned. In the following part, each organism is presented separately, together with the nanosystems developed as potential infection treatments; the results of the related studies and the potential of the various nanosystems as antimicrobial therapeutics are finally discussed. 

## 2. Brief Description of Nanosystems or Nanoparticles Used as Therapeutics

**Figure 1:**
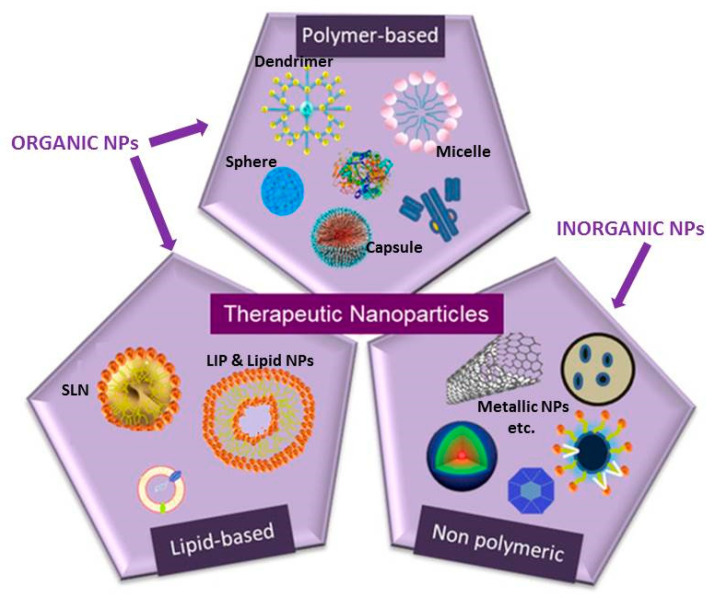
Main types of nanoparticles used as drug carriers (the figure was adapted with permission of [2]. Copyright MDPI, 2020).

The NPs that are used as drug carriers are categorized as organic and inorganic (depending on their composition), and organic NPs (based on their composition) can be categorized as lipidic or polymeric NPs (Figure 1) [2]. The literature searches performed were focused on liposomes (LIPs) or lipid-based NPs and polymeric or other types of NPs. Inorganic, mainly metallic NPs were only included if the NP was associated with drugs or therapeutic modalities. A brief description of the main types of NPs that are currently considered for drug delivery applications follows.

### 2.1. Liposomes and Lipid-Based NPs

#### 2.1.1. Liposomes

As seen in Figure 1, LIPs are vesicular structures composed of phospholipids (PLs) and cholesterol (Chol). PLs and Chol spontaneously form bilayers that eventually “close” to form round-shaped vesicles when hydrated, encapsulating volumes of the hydrating solution. LIPs are categorized depending on their size and number of lamella, as multilamellar vesicles (MLVs), and large or small unilamellar (one bilayer) vesicles (LUVs or SUVs, respectively) [2,4,5]. LIPs may encapsulate/incorporate hydrophobic and/or hydrophilic drugs. The LIP type that is preferentially used as a drug carrier today is the SUV, with diameters between 30 and 200 nm. In addition to having nano dimensions, LIPs should have the capability to circulate in blood (following administration), avoiding rapid uptake by macrophages of the reticuloendothelial system (RES). For the latter purpose, polyethylene glycol (PEG) is used to coat the LIP surface and make it more hydrophilic, sterically stabilizing the vesicles and thus minimizing LIP/plasma–lipoprotein interactions; as a result, the LIP blood circulation half-life is increased [2,3,4,5]. In addition to PEG, the LIP surface can be easily modified with different types of targeting ligands (peptides, monoclonal antibodies, small molecules, aptamers, etc.) for targeted delivery of the LIPs (and the LIP loads) to specific tissues or cell types, where receptors for the selected ligands are over-expressed. Multi-targeted LIP and multifunctional ones (where physical methods are applied to enhance their targeting potential) are currently being exploited as therapeutic systems [3].

The main advantage of using LIPs as drug carriers, in addition to their capability to be loaded with practically any drug and the possibility to modify their size and surface properties on demand, is their excellent biocompatibility and their non-toxic nature.

Compared to polymeric NPs, LIPs are usually degraded faster and thereby cannot be used to sustain the release of active substances for prolonged time periods, such as 6 months or more. Hybrid liposome/polymer systems may be possible solutions, in the latter cases [2,3,4,5].

#### 2.1.2. Other Types of Lipid NPs

Solid lipid NPs (or SLNs) and cationic LIPs or lipid NPs are other types of DDSs that are composed of lipids but have different structures than LIPs in most cases [2,4,5]. SLNs are lipidic spheres that are stabilized by different types of surface-active ingredients. Depending on the amount and type of surfactants used, SLNs may be considerably more toxic than LIPs [2]. Cationic LIPs or lipid NPs may have similar structure with LIPs (bilayer membrane and aqueous core), or they may consist of a lipid core (comprised of oligonucleotidecationic lipid complexes); such lipid NPs are currently particularly popular due to the recent approval of mRNA vaccines against the SARS-CoV-2 virus pandemic [5].

### 2.2. Non-Lipidic NPs

Non-lipidic NPs can be categorized as polymeric NPs and other NP types (which are mostly inorganic). More details for each type are mentioned below.

#### 2.2.1. Polymer-Based NPs

Polymeric NPs are sphere-shaped NPs, composed of biocompatible and biodegradable polymers. Their size (diameter) usually ranges between 10 and 300 nm. They may be composed by natural polymers, such as albumin, gelatin, chitosan, dextran, etc., or by biodegradable synthetic polymers, such as polylactic acid (PLA), polyglycolide (PGA), polyaspartic acid polyethyleneimine (PEI), poly(L-lysine), and poly-alkyl cyanoacrylate, or the copolymer poly lactic-co-glycolic acid (PLGA). They can be spheres having solid matrix cores, or capsules or polymerosomes having liquid cores in solid polymeric shells. PLGA NPs possess the capability to entrap hydrophilic and hydrophobic drug molecules and to control release for prolonged time periods [2].

As in the case of LIPs, the NP diameter, surface properties, and stability are all important parameters that determine their potential as drug carriers. Polymeric NPs can be also coated with PEG in order to prolong their circulation in blood. The most important requirements are that the NPs have low toxicity and that they are biocompatible and biodegradable. Between the various polymeric NP types considered, albumin, PLGA, and chitosan NPs are the most frequently studied [2].

In comparison to LIPs, polymeric NPs offer more ways to control the release of accommodated drugs, since release can be tuned by controlling the NP physicochemical properties (size, structure), as well as the molecular weight of the polymer (degradation rate). Indeed, by using polymers with different MW, or copolymers with different compositions, one can easily tune the release of NP-associated drugs, depending on the particular application [2].

#### 2.2.2. Other Types of NPs

Between the other types of NPs considered for drug delivery applications, we should mention dendrimers and micelles (from the organic NP types), as well as some non-polymeric inorganic NPs, such as silica NPs and various types of metallic NPs, such as silver, gold, and iron oxide NPs [2]. While metallic NPs have intrinsic antimicrobial activity when used alone or in combinations [6,7], we will focus only on their applications as drug carriers. More details about the structures, properties, and methods of manufacturing of the various types of NPs can be found elsewhere [2,3,4,5,6,7].

## 3. Antimicrobial Therapeutics and Other Actives Used against Infections

Concerning the therapeutic modalities that may be combined with NPs for the treatment of infections, we provide in the next section a brief description of antimicrobial drug (antibacterial) categories. In addition, bacteriophages and other therapeutic agents (most of them derived from phages or bacteria) are introduced.

### 3.1. Antimicrobial Drug Categories

Antimicrobials and more specifically antibacterial agents have been classified according to their targets in the bacterial cell. They target various bacterial functions such as cell wall synthesis, cell membrane structure, as well as translation, transcription, and DNA synthesis (Table 1). These targets are different or absent in eukaryotic cells of humans; thereby, antibacterial agents are not active against human cells.

The wide use of antimicrobials has reduced morbidity and mortality worldwide. However, frequent use of wide-range antibacterials in the clinical practice has led to an emergence of multidrug-resistant pathogens, causing mainly nosocomial infections. The most important multidrug-resistant bacteria include methicillin-resistant *Staphylococcus aureus*, vancomycin-resistant Enterococci, and extended-spectrum β-lactamase or carbapenemases-producing Gram-negative rods (e.g., *Escherichia coli*, *Klebsiella pneumoniae*, *Acinetobacter baumannii*, and *Pseudomonas aeruginosa*).

Four major mechanisms mediating potential bacterial resistance toward antimicrobial therapeutics have been identified: (a) production of drug-inactivating enzymes by the bacteria (e.g., β-lactamases, extended-spectrum β-lactamases, carbapenemases); (b) modification of drug target in the bacterial cell (e.g., methylation of 23S rRNA results in resistance toward erythromycin); (c) reduction of drug permeability and drug uptake by cells or intracellular compartments (e.g., changes in porins of Gram-negative bacteria); (d) bacteria encoding efflux pumps; this is a more complex mechanism by which bacteria can actively export drugs from the intracellular to the extracellular space. The genetic information for these mechanisms may be located on the bacterial chromosome (mutations), or on mobile genetic elements such as plasmids, transposons, or phages. Resistance genes can be transferred among bacteria located in the normal micobiota flora, rendering a susceptible population resistant after the suscessful acquisition of such new genetic elements [8]. The three principles of antimicrobials’ stewardship include reduction of inappropriate use of antibiotics, targeted treatment preferring narrow-spectrum drugs, and limitation of any adverse effects. Targeted treatment requires a prompt laboratory diagnosis and specific antibiotic therapy, and it offers the best safety profile for patients. For this purpose, narrow-spectrum antibiotics, phages, and nanosystems for targeted drug delivery are being increasingly considered in the recent years.

### 3.2. Bacteriophages as Antimicrobials

**Figure 2:**
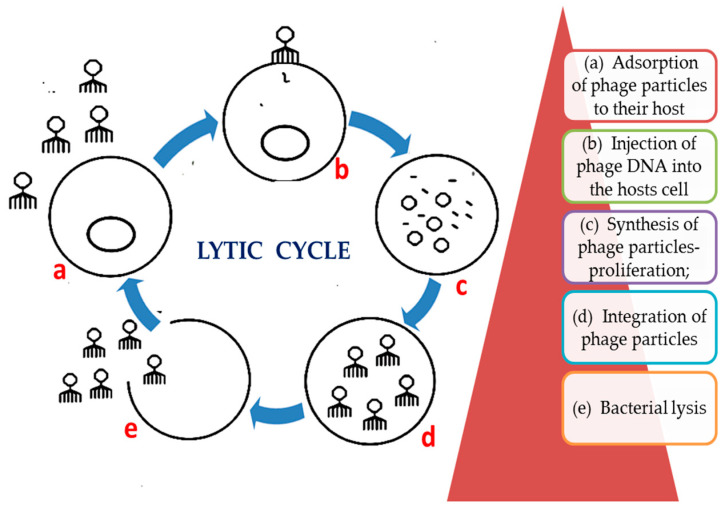
The lytic cycle of bacteriophages.

In 1915, Twort and D’Herelle discovered bacteria-infecting viruses in a dysentery bacilli culture; they were called “bacteriophages” or “phages”. Ιn 1919, phages were used to treat *Shigella dysenteriae* [9]. Nucleic acid (DNA or RNA) enclosed in the phage capsid can be transfered into the bacterium and use its metabolic machinery to reproduce after the capsid is adsorbed to the bacterial cell wall. This lytic life-cycle leads bacterial death, making phages natural antimicrobials (Figure 2) [10,11]. The number of bacteriophages on Earth is estimated at 10^31^ particles, which is ten times more than their bacterial counterparts, and they are spread in the entire biosphere, maintaining the balance of the ecosystem, including the human body [12].

Bacteriophages have been used as antimicrobial therapeutics mainly in Eastern Europe and Russia [13]. Recently, the interest in investigating phages and their lytic proteins (separately), as antibacterial agents, has been renewed due to the increased rate of multidrug-resistant bacteria that cause nosocomial infections. Additionally, endolysins and phage cocktails that have ability to degrade polysaccharide biofilms have been considered for the treatment of implant-associated infections [10,11,12,14]. Phages show specificity for their hosts; therefore, each phage has a narrow spectrum of bacterial species that it infects, despite the antibiotic resistance determinants that the bacterium carries. For example, in the case of *Staphylococcus aureus* infections, both strains, methicillin-resistant and methicillin-sensitive, are susceptible to phages [13,15,16,17]. Phages only replicate in the infection site where their host is located, and thereby, they do not cause side effects. Μoreover, bacteria that confer resistance to certain phages can be susceptible toward others. This finding led the investigators to use phage cocktails for therapy or decolonization [13,15,16,17]. Bacteriophage/antibiotic combinations have been also used for the treatment of biofilm-forming infections; however, further studies are needed in order to better elucidate the potential of such combinations [18]. The successful use of phage cocktails against multi-drug resistant Gram-negative pathogens, such as Carbapenem-resistant *Pseudomonas aeruginosa*, *Acinetobacter baumannii*, *Salmonella*, and an extended spectrum β-lactamase producing *Escherichia coli* and *Klebsiella pneumoniae*, has also been reported [19,20,21]. An ideal antimicrobial agent in addition to its antibacterial spectrum should (preferably) not cause bacterial resistance. However, bacteria can become resistant to phages. Resistance mechanisms are observed when interruptions during the phage life cycle occur; bacteria may block phage receptors, preventing adsorption (phage adsorption inhibition), or they may block phage injection, or the bacterium may protect its DNA from a phage-driven restriction-modification by methylating specific DNA sequences [22].

### 3.3. Other Therapeutic Substances

The worldwide increased cases of bacterial resistance have led investigators to consider novel therapeutic options. For example, the buildup of resistance to mupirocin by methicillin-resistant *S. aureus* (MRSA) led to the use of new antimicrobial therapies, such as bacteriophages, omiganan pentahydrochloride, polyhexanide, ethanol, tea tree oil, lysostaphin, probiotics, and honey [23]. Other alternative therapies of MRSA infections include antimicrobial peptides produced by Staphylococcus, called bacteriocins, which inhibit the growth of S. aureus, as well as plant- and animal-derived compounds, cationic antimicrobial peptides, photodynamic therapy, and bacteriophages (as mentioned above) [13].

Another approach (for MRSA treatment) is to use phage lytic proteins (endolysins and virion-associated peptidoglycan hydrolases). As potential antimicrobials, phages and lysins have some important common features, such as the ability to kill antibiotic-resistant bacteria, narrow antibacterial range, and lack of toxicity to mammalian cells [11]. Over the past two decades, lysins have been developed and successfully tested in animal models to treat patients with MRSA bacteremia or endocarditis [11,12]. Another lysin (PlySs2), derived from a *Streptococcus suis* phage, has a wide lytic activity against various species of Gram-positive bacteria. In a bacteremia mouse model of mixed MRSA and *S. pyogenes*, one dose of PlySs2 protected 92% of mice, with no resistance developed [24]. Lysins have also been used in combination with other agents for enhanced antibacterial effect. An endolysin, Ts2631, from an extremophilic bacteriophage that infects *Thermus scotoductus* was active against multi-resistant clinical strains of *A. baumannii*, *P. aeruginosa*, and *Enterobacteriaceae* (*E. coli*, *C. freundii*, *C. braakii*, *K. pneumoniae*, and *Enterobacter cloacae*), especially in combination with EDTA. This combination reduced all pathogens of the *Enterobacteriaceae* family, and particularly the multidrug-resistant *C. braakii*, to levels below their detection limits [25].

Another strategy for the treatment of vancomycin-resistant enterococcal (VRE) infections is fecal microbiota transplantation (FMT). FMT has been applied for treatment of intestinal infections for centuries and was first described by a traditional Chinese medicine doctor (284–364 BC). The first clinical trial of FMT reported its superiority to vancomycin [26]. FMT is used to treat colonization and infection by *Clostridium difficille* and VRE and to recover gut microbiota after antibiotic overuse.

## 4. Nanosystems as Antimicrobial Treatments of Infections

**Figure 3:**
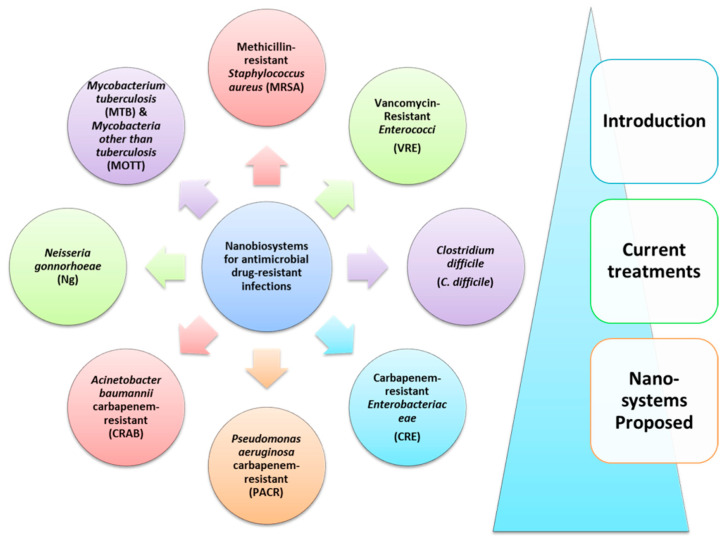
Pathogens investigated for proposed nanosystem treatments for infections.

This section is divided in subsections for each specific pathogen. Each subsection includes an introduction about the pathogen and its related infections, the mechanisms of resistance buildup, and the types of conventional therapeutic treatments currently used, including treatments with phages (if applying). After that, the various cases of antimicrobial nanosystems that were found in the literature searches are discussed in more detail (Figure 3).

### 4.1. Methicillin-Resistant Staphylococcus Aureus (MRSA)

#### 4.1.1. Introduction about MRSA Infections and Treatment Approaches

*Staphylococcus aureus* is one of the most important human pathogens and the etiologic agent of a variety of infections including bacteremia, endocarditis, pneumonia, skin, and soft tissue as well as prosthetic device-related infections [27]. Over the last decades, β-lactam antibiotics played an important role for the treatment of *S. aureus* infections. However, *S. aureus* producing β-lactamases emerged soon after the introduction of β-lactams. Moreover, the acquisition of a mobile genetic element, the staphylococcal cassette chromosome SCC*mec*, gave rise to methicillin-resistant *S. aureus* (MRSA) strains that are also resistant to all β-lactams [28].

Phage treatment was especially useful in the absence of other options during the pre-antibiotic era; this is again true, due to an overwhelming increase in antibiotic resistance [9]. This type of therapy relied on phages from nature, which lyse bacteria at the site of infection. However, advances in biotechnology led to novel strategies using bioengineered phages and purified phage lytic proteins [9]. A potential application of phages is the eradication/reduction of MRSA nasal colonization [29].

Phages have been tested in vitro against MRSA with positive results. An in situ model study of hand washing using a solution of phage K resulted in a 100-fold reduction of staphylococcal numbers on human skin [30]. Bacteriophages have been tested in vitro against planktonic bacteria and biofilms with good results, especially against the planktonic bacteria [16,31]. A combination of phage and linezolid was tested as a method to prevent MRSA colonization on an orthopedic implant surface, and it resulted in reduced bacterial adherence without significant emergence of resistant mutants [32]. In another case, the combination of phages with sub-inhibitory concentrations of antibiotics exhibited synergistic effects toward MRSA biofilm [33].

The antimicrobial properties of phages have also been reported in several in vivo studies, alone or combined with antibiotics [34]. Phages are also useful for fomite decontamination [35].

#### 4.1.2. Nanosystems for Treatment of MRSA

Various types of NPs loaded with antimicrobial agents are used as alternative treatments of MRSA infections, and have demonstrated therapeutic advantages compared to conventional formulations [36]. Furthermore, NPs that can deliver antibiotics to intracellular pathogens (such as MRSA) can treat persistent and/or re-occurring infections [37]. Several examples of various NP formulations used against MRSA are mentioned below.

##### Lipid-Based NPs for Treatment of MRSA

For lipid-based NPs against MRSA, most studies are about vancomycin (VAN) incorporated in different LIP types. VAN-loaded conventional and PEGylated LIPs have been developed [38]. Although free VAN was unable to kill macrophage MRSA, conventional VAN LIPs resulted in sufficient VAN concentrations inside macrophages and exerted a significant MRSA bactericidal effect [39]. PEGylated VAN LIPs were used against MRSA pneumonia in high-risk patients; other novel anti-MRSA VAN LIPs, such as inhalable LIPs, were also studied [40]. The encapsulation of VAN into LIPs improved its antistaphylococcal activity in vitro and in vivo compared to free drug injections [41]. In an in vivo study (murine model), PEGylated VAN LIPs significantly prolonged the drug blood circulation time and increased the drug deposition in lungs, liver and spleen, while reducing the drug concentration in kidneys and thereby its nephrotoxicity [42,43]. Bacterial toxins were used to activate drug release from VAN-LIPs that were stabilized with gold NPs, and the LIP-encapsulated VAN was released within 24 h when in the presence of MRSA bacteria, leading to bacterial growth inhibition [44].

More recently, advanced types of LIP-VAN were proposed. Fusogenic VAN LIPs had enhanced antimicrobial activity against mature *S. aureus* biofilms, since LIPs could penetrate the bacterial biofilm [45]. The targeted delivery of VAN by novel pH-responsive LIPs consisted from two-chain fatty acid-based lipids, and they confered superior in vitro activity compared to free drug [46]. Several types of lipid–polymer hybrid NPs were loaded with VAN for the selection of combinations with optimal encapsulation and activity [47]. In another in vitro study, fusogenic LIPs loaded with antibiotics (VAN, methicillin, ampicillin) and functionalized with cell-penetrating peptides (Tat) were highly effective in decreasing the minimum inhibitory concentrations (MICs) of the antibiotics, especially for methicillin [48]. The delivery of VAN by targeted LIPs using biocompatible pH-sensitive lipids conferred increased antibiotic release, in vitro antibacterial activity against MRSA, and in vivo reduction of MRSA growth in infected mice (compared to mice treated with free drug) [49]. In vivo VAN LIPs treated mice (skin infection model) had significantly lower amounts of MRSA compared to mice treated with free VAN [50]. Collagen mimetic peptide tethered VAN-loaded LIPs were hybridized to collagen-based hydrogels for the treatment of MRSA infections, and enhanced in vitro antibacterial effect was demonstrated. The optimal formulation successfully treated MRSA infected wounds in vivo, even after re-inoculation with bacteria [51].

Other LIP-associated drugs were also tested against MRSA, including azithromycin, chloramphenicol, daptomycin clarithromycin, and others. Azithromycin LIPs were used for the local treatment of MRSA skin infections efficiently inhibited MRSA growth and were more efficient in preventing biofilm formation and reducing staphylococcal MIC and minimum bactericidal concentration (MBC) compared to free drug [52]. A novel antibacterial peptide DP7-C and azithromycin LIPs combined with DP7-C-augmented the antibacterial effect of the drug in vivo (mice infected by MRSA) with fewer side effects [53]. Deoxycholic acid (DA)-containing LIPs (which may be considered as elastic LIPs), that were loaded with Chloramphenicol showed increased follicular drug uptake and enhanced antibacterial activity compared to Chloramphenicol alone. In vivo, the DA LIPs caused negligible skin toxicity [54]. A LIP-in-hydrogel system for the dermal delivery of Chloramphenicol was tested ex vivo and showed sustained drug release and limited skin permeation, whereas the antimicrobial activity of Chloramphenicol was similar or enhanced compared to treatment with free drug [55]. Daptomycin LIPs exhibited specific binding to MRSA and good targeting of the drug to MRSA-infected lungs in a mouse model of MRSA pneumonia [56]. Wheat germ agglutinin-modified LIPs encapsulating clarithromycin inhibited the formation of biofilms and enabled the disassembly of pre-existing biofilms. MRSA colony-forming units in kidneys and spleen were significantly decreased compared to those observed with free antibiotic or non-targeted LIPs [57]. Chitosan-coated deformable LIPs containing dicloxacillin exhibited a slow drug release behavior and enhanced activity [58]. LIP systems with longer alkyl-gallates reduced oxacillin’s MIC against MRSA below the antibiotic breakpoint for resistance [59]. Soya ethylmorpholiniumethosulfate (a cationic amphiphile) in LIPs or nanoemulsions, reduced skin infection, MRSA load, and inflammation in infected mice [60].

Natural or semisynthetic constructs loaded in LIPs have been proposed as MRSA treatments. Semisynthetic constructs from nisin, an antibiotic produced by *Lactococcus lactis*, inhibited bacterial growth and were active against clinical MRSA and VRE isolates [61]. LIPs loaded with biosurfactants from *Lactobacillus* were considered against MRSA biofilms. LIPs were non-cytotoxic and prevented biofilm formation by MRSA isolates [62]. Cinnamon oil LIPs exhibited successful antibacterial performance on MRSA and staphylococcal biofilms. LIPs prolonged the period of therapeutic plasma drug concentrations and improved cinnamon oil stability, augmenting its activity against MRSA biofilms [63]. The encapsulation of b-lapachone (a natural naphthoquinone with low solubility) into LIPs did not lower its antibacterial activity against MRSA and improved its antifungal action against *Cryptococcus neoformans* [64]. NanoLIPs loaded with plant-derived epigallocatechin gallate were tested in vitro and in vivo on burned mouse skin infected by MRSA; cationic nanoLIPs were more effective against MRSA [65]. Silymarin-LIPs had lower MICs against MRSA, higher killing rates, and better in vivo survival rates when applied on MRSA-infected burn wounds of mice compared to the free compound [66]. LIPs loaded with oleic acid were found to rapidly fuse into the bacterial membranes, significantly improving the potency of oleic acid against MRSA. They were highly effective against MRSA skin infections in mice, preserving the integrity of the infected skin [67].

Tailored LIPs (without antimicrobial drugs) were effective against infections, including MRSA. LIPs composed of sphingomyelin sequestered cytolytic toxins secreted by USA300 *S. aureus* and prevented erythrocyte peripheral blood mononuclear cell and bronchial epithelial cell necrosis, and also hemolysis. In vivo, the LIPs significantly decreased tissue dermonecrosis [68]. LIP-bound toxins (inactive against mammalian cells) were used as decoys to sequester bacterial toxins. The LIPs rescued mice from *S. aureus* and *S. pneumoniae* septicemia; untreated mice died [69].

Antimicrobial photosensitizing agents loaded in LIPs, were also considered as a strategy against MRSA [70]. Hematoporphyrin was encapsulated in LIPs and micelles, and combinations of the latter completely eradicated bacteria at lower doses than those required for the free photosensitizer; micelles had the best results [71]. *m*-tetrahydroxyphenylchlorin was encapsulated in cationic LIPs and evaluated against MRSA [72]. An antimicrobial peptide, named WLBU2 and temoporfin, a potent generation II photosensitizer were entrapped in LIPs, and more temoporfin was delivered to bacteria while LIPs killed all MRSA in vitro [73]. Combinations of porphyrin-type photosensitizers with polycationic LIPs enhanced their antibacterial effect, as LIPs disorganized the bacterial wall, enhancing photosensitizer permeability [74].

An oleic acid and gentamicin combination, in free and LIP forms, was compared to free VAN. LIPs exhibited enhanced antibacterial activity compared to the free drugs and to VAN [75]. VAN and beta-lactams combinations improved the clinical outcome in patients with MRSA infections. VAN LIPs reduced the drug’s MIC by 2-fold compared to free drug; VAN+cefazolin LIPs further decreased the MIC. Long circulation and reduced kidney clearance of LIPs resulted in higher efficacy and reduced nephrotoxicity [76]. Daptomycin–clarithromycin LIPs were also tested, and the combination was an effective and less toxic treatment for MRSA infections [77].

VAN-loaded LIPs coupled with lysostaphin as the targeting ligand and the targeted LIPs suppressed bacterial infection in vitro and in vivo more effectively than equal doses of untargeted LIPs [43].

The entrapment of phage cocktails inside LIPs is also considered as an alternative treatment of persistent bacterial infections, as increased phage persistence in vitro and in vivo after application in diabetic infected mice has been observed [78]. Nanostructured lipid-based carriers, i.e., transfersomes, are effective as transdermal delivery systems of MRSA phage cocktails as shown from treating rats with soft-tissue infections [15].

Concerning non-conventional LIPs, novel anionic LIPs loaded with anti-*mecA* phosphorothioate oligodeoxynucleotide and polyethylenimine, to target *mecA*, restored the susceptibility of MRSA to β-lactam antibiotics. In vitro, the anti-*mecA* complex decreased *mecA* expression and inhibited MRSA growth. In vivo, the MICs of five antibiotics against clinical isolates of MRSA were reduced (to values in the sensitivity range), and mice were rescued from MRSA-caused septic death by downregulating *mecA* [79].

Transcription factor decoys (oligonucleotides that inhibit gene transcription) were loaded in cationic lipid NPs with VAN and significantly decreased MRSA viability (in cultures) compared with VAN alone with very low levels of cytotoxicity and hemolysis in vitro [80]. Oxacillin cationic nano-lipid carriers (NLC) exhibited synergistic MRSA eradication by lowering MIC and biofilm thickness. Topical administration significantly reduced cutaneous infection in mice and improved skin barrier function and architecture [81]. Monolaurin–lipid nanocapsules combined with a plectasin derivative demonstarted promising results against S. aureus, including MRSA [82]. Others developed a new lipid–dendrimer hybrid NP system to effectively deliver VAN against MRSA infections [83].

##### Non-Lipidic NPs for Treatment of MRSA

Between the non-lipidic NPs to treat MRSA infections, metallic and other types of inorganic NPs have been used, as well as organic NPs (Figure 1).

Starting from the inorganic NPs, silver NPs (AgNPs) are potential antimicrobial agents. A new treatment for MRSA infections could be the combination of AgNPs with blue light and antibiotics such as amoxicillin, azithromycin, clarithromycin, or linezolid [84]. Other studies focused on the conjugation of AgNPs with other antimicrobials such as cephradine and vildagliptin [85], rifampicin [86], simvastatin [87], or cefotaxime [88] and showed their enhanced antibacterial activity.

Combinations of AgNPs and antibiotics (VAN, rifampin, gentamicin, and levofloxacin), significantly enhanced the antibacterial effect both in vitro and in vivo in infected rats [89]. Gentamicin-loaded silk/nanosilver composite scaffolds were tested against MRSA to treat osteomyelitis in rats with superior antibacterial outcome [90].

Gold NPs combined with gentamicin provided continuous drug release over days. Glutathione was a better coupling agent than cysteine, allowing higher loading and resulting in lower MIC values [91]. Daptomycin-loaded gold nanocages conjugated to antibodies to target two different *S. aureus* lipoproteins effectively killed MRSA in biofilms [92].

Other inorganic NPs were tested, such as magnetic NPs bonded to oxacillin that was tested on a local infection rat model, finding significantly lowered bacterial counts and inflammatory reactions in muscles and lungs [93]. Silica–gentamicin delivery systems were efficient against planktonic MRSA and *E. coli* biofilms, with a non-significant increase of mortality in zebrafish embryos [94]. Selenium NPs (SeNPs) combined with ampicillin, oxacillin, and penicillin showed strong effects against biofilms [95]. VAN-loaded aragonite NPs from cockle shells exhibited high antibacterial effects against MRSA [96].

On the intephase of organic/inorganic NPs, chitosan–magnetic NPs were synthesized and loaded with streptomycin, resulting in enhanced activity toward MRSA [97].

Going to organic NPs, ceftriaxone-loaded chitosan NPs were assessed on a neutropenic mouse model with good results; a 41% decrease in MRSA was observed [98]. NP capsules with a core from peppermint oil and cinnamaldehyde acted as potent antimicrobial agents with positive results against biofilms while also promoting fibroblast proliferation in a mixed bacteria/mammalian cell system, having potential applications in wound healing [99]. Polyacrylate-NP antibiotics, or *nanobiotics*, enhanced the antimicrobial activity of water-insoluble antibiotics such as *N*-methylthio β-lactams [100] When penicillin was loaded in the latter polyacrylate NPs, equipotent in vitro antibacterial properties against MSSA and MRSA were demonstrated, but the particle had indefinite stability toward β-lactamases [101]. Kalita et al. reported the enhanced anti-MRSA activity of chloramphenicol loaded in poly-ε-caprolactone/pluronic composite NPs in vivo on a MRSA-infected burn-wound animal model [102].

Cationic PLGA-NPs reduced the MIC required for VAN to inhibit the growth of planktonic MRSA and biofilm formation; thus, the combination could be an alternative treatment of MRSA infections [103]. Positively charged clindamycin-loaded PLGA-PEI-NPs bind to the MRSA surface, enhancing the bactericidal efficacy of the drug compared to negatively charged PLGA-NPs. Furthermore, they significantly accelerated the healing and re-epithelialization of MRSA-infected wounds in a mouse model, while being harmless to fibroblasts [104].

Carvacrol, a component of essential oils, was loaded in site-specific NPs incorporated into a hydrogel matrix to facilitate dermal delivery. Carvacrol release from NPs was faster in the presence of bacterial lipases, highlighting the NPs potential for triggered delivery; NPs showed higher activity than free drug in an ex vivo skin infection model [105]. VAN-loaded polymeric NPs were found promising as ocular infection treatments after in vivo application to albino rat eyes [106]. VAN-loaded NPs that consisted of various polymers were highly active against intracellular pathogens including MRSA due to an efficient cellular uptake of VAN. NPs rapidly accumulated in the liver and spleen, which were the target organs of intracellular infection [107].

Table 2 summarizes the different types of nanosystems developed for the treatment of MRSA.

### 4.2. Vancomycin-Resistant Enterococci (VRE)

#### 4.2.1. Introduction to VRE Infections and Current Treatment Approaches

Enterococci are widespread opportunistic pathogens and frequent causes of hospital infections. They are part of the normal flora of animals, colonizing the gastrointestinal tract. Found in sewage water, they survive in other niches, such as the oral cavity, skin, and genitourinary tract. Among various enterococcal species, *Enterococcus faecalis* and *Enterococcus faecium* are mostly associated with human diseases. They cause different types of infections, such as bacteraemia, urinary tract, surgical wound infections, and endocarditis [108].

Enterococci express intrinsic resistance to aminoglycosides, ampicillin, and glycopeptides. Resistance to high-level beta-lactams is caused by the mutation or overproduction of penicillin binding protein 5 (PBP5), while low-level aminoglycoside resistance is associated with a slow uptake of drugs. Acquired resistance to antibiotics, including high-level aminoglycosides, chloramphenicol, and glycopeptides is a major healthcare problem. Since their appearance (1988), VRE have been spreading rapidly worldwide [108]. VRE are important nosocomial pathogens that commonly affect critically ill patients, especially those that receive prolonged antibiotic treatments. VRE are common problems in intensive care units outnumbered only by coagulase-negative staphylococci, as they frequently cause bloodstream infections in high-risk patients [109]. VRE have remarkable genetic plasticity, allowing them to acquire genes associated with antimicrobial resistance, making their eradication very difficult [110].

Treatment options for VRE infections include various antimicrobials; tigecycline, linezolid, daptomycin, quinupristin-dalfopristin, platensimycin, nitrofurantoin, and fosfomycin have been used [110]. Phages could also treat a broad range of antibiotic-resistant enterococcal infections. Several bacteriophages are active against *E. faecalis* strains from oral cavity of root canal infection patients. One phage (SHEF2), with the broadest host range, was able to eradicate *E. faecalis* biofilms formed in vitro on a polystyrene surface but also on a cross-sectional tooth slice model of endodontic infection. Phage SHEF2 cleared a lethal infection of zebrafish in vivo [111]. In another in vivo study, a cocktail of two lytic bacteriophages treated severe septic peritonitis caused by vancomycin-resistant *E. faecalis*, in mice [18]. Bacteriophages from wastewater are also promising tools against pathogens that cause orthopedic implant-associated infections such as *S. aureus*, *E. faecalis*, *E. coli*, and VRE [14].

In dentistry, *E. faecalis*, especially VRE strains that produce biofilm, represent a common threat in recurrent root canal treatment failures. Phage therapy was highly effective against such strains, including biofilm-forming ones [112].

#### 4.2.2. Nanosystems for Treatment of VRE Infections

Nanosystems for the treatment of VRE infections are summarized in Table 3.

As seen, few cases of inorganic and even fewer of organic NPs (3 and 2) to treat VRE infections were reported. Staring from inorganic NPs, the combination of metallic ZnO NPs (loaded) with antibiotics was effective against the resistant bacteria (VRE) in a dose-dependent manner, reducing the antimicrobial MICs [113]. Magnetic NPs, loaded with vancomycin (VAN) and combined with receptor-specific ligands in order to induce polyvalent interactions, were proposed as a promising and sensitive tool for the detection of pathogens, including VRE [114]. In another case, VAN activity against VRE and methicillin-resistant *Staphylococcus epidermidis* was enhanced when VAN was conjugated to silver NPs [115].

Two reports of organic NPs against VRE were found. In the first, LIPs combined with six different fatty acids and four cholesteryl esters were tested against isolates from hospital settings, including VRE. Selected fatty acids and cholesteryl esters in LIPs exhibited antibacterial activity and also enhanced the activity of antibiotics [116]. In the second case, NPs consisted of block copolymer DA95B5 effectively removed biofilms of various clinically relevant multidrug-resistant bacteria, including MRSA and VRE, in a murine wound model. NPs penetrated into biofilm and promoted the gradual dispersal of bacteria. Such NPs could also be applied to hydrogel dressings for other applications against bacterial biofilms [117].

### 4.3. Clostridium Difficile (CD)

#### 4.3.1. Introduction in CD Infections (CDI) and Current Treatment Approaches

*Clostridium difficile* is an opportunistic, Gram-positive bacterium frequently associated with intestinal infections. It is the most common cause of hospital-acquired diarrhea, which has increased incidence over the last decades, due to broad-spectrum antibiotics [118]. The pathogen is non-invasive, and virulence is mostly due to enzymes, such as collagenase and hyaluronidase, as well as toxins, which damage the epithelial cell cytoskeleton, leading to the disruption of tight junctions, followed by fluid secretion, neutrophil adhesion, and local inflammation. The result is a breakdown of the gut barrier integrity and loss of its functionality [118]. *C. difficile *produces two important types of toxins, A and B, which are both enterotoxic and cytotoxic. The epithelial barrier damaging leads to the translocation of commensal bacteria and the influx of inflammatory cells [119]. After the introduction of antibiotics and their widespread use, the role of *C. difficile* in the pathogenesis of large intestine disease has increased. *C. difficile* is intrinsically resistant to several antibiotics and outcompetes other susceptible gut flora, proliferating rapidly and producing toxins [120].

*C. difficile* infection is usually treated with metronidazole. This was the first-line drug in non-severe CDI, while VAN has been the drug of choice for severe CDI. Fidaxomicin, available since 2011, is a macrocyclic bactericidal antibiotic with high efficacy against *C. difficile*, not affecting the physiological intestinal flora [121].

Alternatively, *C. difficile* phages can alter virulence-associated phenotypes such as toxin production by interfering with bacterial regulatory circuits. Phage genomes often contain multiple regulatory genes, having a complex and often subtle impact on the host. The main virulence factors of *C. difficile*, the exotoxins TcdA and TcdB, are encoded by the PaLoc, which is a 19.6-kb pathogenicity locus [118] that is thought to originate from an ancient prophage. This locus shares a number of features with phages, in particular the *tcdE* gene encoding a phage-like holin involved in toxin secretion. Prophages seem to impact the biology of *C. difficile* in subtle ways, depending on the host’s genetic background [122].

The minimal adverse effect phages have on the gut microbiota results in the prevention of further dysbiosis during CDI. Furthermore, phages replicate in a self-limiting manner at the localized infection site; thus, their effective dose is amplified [123]. A third useful attribute of phages is their ability to penetrate into complex biofilm environments found in *C. difficile*-associated pseudomembranous plaques [124]. The activity of seven phages against clinical strains of *C. difficile* was tested, and phage combinations reduced colonization and symptoms and extended lifespan in a hamster model of CDI, supporting the potential application of phage coctails [125].

Fecal microbiota transplantation (FMT) was used on the “Zurich Patient”, who after recurring CDI episodes experienced clinical cure within 2 weeks of FMT. By applying metagenomic and 16S rRNA gene sequencing, a study focused on changes of bacterial and viral populations in patient and donor feces for 4.5 years after FMT, and it concluded that the virome was mainly composed of *Caudovirales* bacteriophages [126]. Studies to find if bacteriophages were associated with restoration of the intestinal flora after FMT concluded that there was a correlation between donor viral richness and patients response to FMT [127].

#### 4.3.2. Nanosystems for Treatment of CDI

A few reports were found about nanosystems to treat CDI (Table 4), and they are described below.

Iron oxide NPs (IONPs), chitosan NPs, LIPs, andcationic lipid NPs were used against CDI. VAN-conjugated IONPs that specifically bind to *C. difficile* spores and exhibit antimicrobial activity were developed. They successfully delayed the germination of spores and also inhibited (by ≈50%) vegetative cell outgrowth after 48 h incubations. They also inhibited the interaction of spores with HT-29 intestinal mucosal cells in vitro. In vivo (in a murine model), the IONPs significantly protected mice from *C. difficile* compared to free VAN (reducted intestinal inflammation and superior mucosal viability were demonstrated) [128]. In another in vitro study, metronidazole-loaded particles were prepared and coated with chitosan for increased mucoadhesive properties. Optimized NPs had high drug load and good particle retention on porcine mucosa. Dry coating of the chitosan-coated microparticles with hydrophilic fumed silica improved their dispersibility, as indicated by visual observation and dissolution studies [129].

Antisense sequences that could downregulate the expression of *C. difficile* essential genes, by blocking mRNA translation, were synthesized as 2′-*O*-methyl phosphorothioate gapmer antisense oligonucleotides (ASO). Antisense gapmers were entraped in cationic bolasomes (LIPs with bile acids), and such gapmers achieved nanomolar MICs against four gene targets of *C. difficile*; the lowest values were for ASOs targeting polymerase genes *rpoB* and *dnaE* [130].

Cecally cannulated horses were utilized to determine the optimal dose of curcumin LIP to reduce bacteria populations, including *C. difficile*, without adversely affecting cecal characteristics. The LIPs had no significant effect on *C. difficile* growth; on the contrary (at high doses), they increased opportunistic bacteria populations [131].

### 4.4. Carbapenem-Resistant Enterobacteriaceae (CRE)

#### 4.4.1. Introduction in CRE Infections and Current Treatment Approaches

*Enterobacteriaceae* include several bacterial species, such as *Escherichia coli* and *Klebsiella pneumoniae*, which are part of the normal human intestinal flora, but they are also frequent etiologic agents of community/healthcare-related infections. These bacteria have the ability to acquire resistance genes, and increased antimicrobial resistance has been observed.

Carbapenems are β-lactam antibiotics active against a wide range of Gram-negative (including *Enterobacteriaceae*) and Gram-positive bacteria. They are very potent against resistant bacteria, including various ESBL (extended spectrum β-lactamases)-producing microbes. Carbapenems are usually the last resort antimicrobials considered as “treatment of choice” against serious infections due to their “concentration-independent killing effect” [132].

Carbapenem resistance in *Enterobacteriaceae* can be developed via several mechanisms: reduced outer-membrane permeability due to the modification of membrane porins, expression loss or shift of porin proteins, enzymatic inactivation of drugs by plasmid-mediated or chromosomal enzymes with hydrolytic activity, and antibiotic efflux through efflux pumps. Risk factors for the acquisition of CRE in healthcare settings include admission to intensive care units (ICU), long ICU stay, critical illness, invasive devices, and previous antimicrobial therapy (including other than carbapenem antibiotics) [133]. The genes responsible for carbapenemase production are often located on plasmids together with other resistance genes that can be exchanged between *Enterobacteriaceae* and other Gram-negative bacteria, resulting in multidrug or extensively drug-resistant strains [134].

Class A KPC, the most common carbapenemases, are endemic in China, India, Saudi Arabia, Greece, Columbia, and Brazil. Τhe first KPC enzyme was isolated in North Carolina, USA, from a *K. pneumoniae* clinical isolate, and they are widely disseminated across the world, causing outbreaks. [135]. In 2008, an NDM-producing *K. pneumoniae* isolate was identified in a Swedish patient (recently been to India, where he acquired a urinary tract infection). Since their discovery, NDM carbapenemases have been reported in Enterobacteriaceae worldwide [136].

Treatment options for CRE infections are limited. Antibiotics that are active against CRE include colistin, tigecycline, and fosfomycin. However, reduced in vivo effectiveness, frequent adverse effects, and rapid development of resistance limits their use. Combinations of two or more antimicrobials can increase the survival of patients with serious infections [137]. There is an urgent need for research and clinical development of antimicrobials to keep up with the evolution of bacterial resistance toward CRE. Another alternative could be the use of bacteriophages.

Between the phage therapy advantages, their specificity to pathogens (in contrast with antibiotics that destroy pathogens and normal microbiota), and their unique characteristic of “auto-dosing” (specific dosing is not required), are particular reasons for which phages were used to treat CRE infections [138]. Bacteriophages that are highly specific to *E. coli* PI-7 (a strain that produces NDM) were isolated from municipal wastewater, and a combination of solar irradiation and bacteriophages successfully reduced the length of the lag-phase for *E. coli* PI-7, from 4 to 2 h. Overall, the combined therapy made bacteria more susceptible [139]. In another case, three commercial bacteriophage cocktails were tested against 70 *E. coli* and 31 *Proteus* isolates (including eight carbapenemase-producers; seven *E. coli* strains positive for NDM, IPM, or OXA, and one NDM-positive *Proteus mirabilis* isolate). Five of the eight strains that produced carbapenemases were susceptible to two phage coctails [21]. Other multi-resistant members of the *Enterobacteriaceae* family were also sensitive to phages such as *Citobacter freundii* [140].

A phage cocktail formulation was evaluated for its ability to specifically kill a KPC-positive *K. pneumoniae* strain at various stages of colonization within a multispecies drinking water biofilm community, demonstrating the potential of phages to control carbapenemase-producing *Enterobacteriaceae* associated with microbial biofilms in healthcare settings [141]. Another cocktail of three bacteriophages isolated from sewage water, which were capable of infecting 150 isolates from three members of the *Enterobacteriaceae* family (*E. coli*, *K. pneumoniae*, and *Enterobacter* species), was tested against multiple bacterial mixtures of the aforementioned species, including strains resistant to meropenem and colistin. A reduction in bacterial load by three orders of magnitude was recorded within 2 h in vitro [142].

#### 4.4.2. Nanosystems for Treatment of CRE Infections

Table 5 summarizes all nanosystems for tretament of CRE infections.

The antibacterial efficacy of oligonucleotide transcription factor decoys (TFD) delivered in SLNs was confirmed in vitro by a TFD targeting the Fur iron uptake pathway in *E. coli*. Biocompatibility was assessed in vitro in epithelial cells and in vivo in *Xenopus laevis* embryo models [143].

Meropenem-loaded SLNs, prepared using hot homogenization and ultrasonication, were optimized by experimental design. Differential scanning calorimetry and X-ray diffraction analysis showed that meropenem was present in amorphous form in the NPs. Controlled release of meropenem from NPs was proven in vitro, and prolonged antibacterial activity against *E. coli* strains was also observed [144].

Non-lipidic NPs were also used for CRE treatment; silver NPs synergisticaly enhanced the antibacterial activity of cefotaxime, ceftazidime, meropenem, ciprofloxacin, and gentamicin against multi-resistant, β-lactamase, and carbapenemase-producing *Enterobacteriaceae*. The NP–antibiotic combinations exhibited enhanced antibacterial activity, with lower MICs (compared with MICs of individual antibiotics and silver NPs alone). The enhanced activity of antibiotic/NP combinations, especially for meropenem, was weaker against non-resistant bacteria. Nevertheless, low, non-cyctotoxic (to mammalian cells) silver-NP concentrations were effective to enhance the activity of antibiotics against multi-resistant bacteria [145]. Combinations of silver NPs (bio-AgNP) with oregano (Origanum vulgare) essential oil (OEO) were tested against various multi-resistant bacteria such as MRSA as well as β-lactamase and carbapenemase-producing *E. coli* and *A. baumannii* strains. Time–kill curves indicated that OEO acted rapidly (10 min), while metallic NPs killed the Gram-negative bacteria in 4 h. Synergistic or additive effects of combined treatments were confirmed by reduced antimicrobial MICs and time of action, suggesting a potential alternative pathway for infections where antibiotics are insufficient [146].

In a study to check whether silver NPs (AgNPs) could exert a synergistic effect when combined with beta-lactams against carbapenem-resistant *E. coli*, bacteria were subjected to combinations of beta-lactam antibiotics, specifically amoxicillin, cefoperazone, and doripenem, and a sub-inhibitory dose of AgNPs. The sensitivity of both tretaments against bacteria expressing NDM5 metallo-β-lactamase was increased by ≈250–1000-fold when combined. In the presence of AgNPs, the resistance due to NDM5 was eradicated, reaching the level of cells not expressing NDM5. Therefore, it can be concluded that AgNPs can completely eradicate resistance produced by NDM5 in vivo when combined with beta-lactams. AgNPs could also exert a synergic effect on the *E. coli* cells harboring NDM5 [147].

Platinum NPs have also been used against carbapenem-resistant bacteria. Plasmid curing (loss of plasmids from host strains due to treatment with various compounds) in carbapenem-resistant *E. coli* strains was demonstrated in vivo by sub-MIC, non-toxic levels of platinum NPs. Moreover, a combination of platinum NPs with meropenem significantly reduced bacterial bioburden in infected zebrafish [148].

Gold NPs of different sizes were loaded with imipenem or meropenem and evaluated against carbapenem-resistant Gram-negative bacteria, as well as against *K. pneumoniae*, *P. mirabilis*, and *A. baumannii* isolates resistant to imipenem and meropenem. Particles with 35 nm diameters achieved the highest enhancement of antibacterial activity of both antibiotics against all the selected isolates. The gold-NPs decreased the MICs of imipenem and meropenem toward bacteria, below the resistance breakpoint for carbapenems, being thus promising systems for reducing bacterial resistance to carbapenems [149].

Chitosan NPs conjugated with meropenem were tested toward six bacterial strains including meropenem-sensitive and meropenem-resistant strains of *K. pneumoniae* and *E. coli*, demonstrating higher antibacterial activities against methicillin-sensitive and methicillin-resistant *S. aureus*, *E. coli*, and *K. pneumoniae*, and showing dramatic improvement of survival and bacterial clearance in vivo (septic rat model of *K. pneumoniae)*, compared to the free drug [150]. The controlled co-release of imipenem/cilastatin drugs and hydrocortisone from a novel chitosan-polyethylene oxide nanofibrous mat has also been investigated [151].

Nanospheres consisting of low molecular weight PLGA or polylactic acid (PLA) were loaded with meropenem. The PLGA-NPs led to two times lower *E. coli* growth compared to the PLA-NPs, while both were biocompatible; the results suggest that PLGA can be used for antibiotic delivery after orthopedic surgeries [152].

### 4.5. Pseudomonas Aeruginosa Carbapenem-Resistant (PACR)

#### 4.5.1. Introduction in PACR Infections and Current Treatment Approaches

Among antibiotic-resistant bacteria, *P. aeruginosa* is a frequent cause of infections, especially for immunocompromised or cystic fibrosis patients. Even though there are geographic differences, infections of multidrug-resistant *P. aeruginosa* have recently spread worldwide. The prevalence is 15 to 30% in several areas [153].

Due to its metabolic plasticity and versatility, *P. aeruginosa* is capable of infecting or colonizing a wide range of ecological niches, aquatic and soil habitats, animals, and plants. As far as multi-resistant strains are concerned, high-risk clones are reported to have disseminated in several hospitals worldwide. There is a clear connection between high-risk clones and horizontally acquired resistance mechanisms; most ESBL- or MBL-producing *P. aeruginosa* isolates can be classified into a few clones, with ST235 being the most frequent, followed by ST111 [154].

As in *Enterobacteriacae*, carbapenemases (KPC, GES-2, MBL) have also been reported in *P. aeruginosa*. Metallo-beta-lactamases (MBLs) pose the majority of such enzymes including IMP, VIM, NDM, SPM, and GIM. These enzymes are mostly encoded on mobile genetic elements (plasmids, integrons and cassettes), which are horizontally disseminated between bacteria. MBLs frequently co-exist in bacteria with multiple β-lactam resistance mechanisms. In addition, genes encoding MBLs are often located on mobile genetic elements that also harbor resistant genes responsible for the production of elements against other types of antimicrobials, such as aminoglycoside-modifying enzymes [155].

Phages have been tested for PACR infection treatment. Phage ØA392 was isolated from hospital sewages and was used in mice infected by an imipenem-resistant strain of *P. aeruginosa*. All mice that were bacteremic after i.p. injections of the strain died within 24 h when not treated [156]. In another in vivo study, *P. aeruginosa* was used to induce bacteremia in streptozotocin-induced diabetic and nondiabetic mice by i.p. injection. The selected strain was resistant against many antibiotics, including imipenem. A single dose of phage showed efficient protection in both diabetic (90%) and nondiabetic (100%) bacteremic mice, proving the potential of phage therapy against multi-resistant *P. aeruginosa* infections [157]. In France, 47 multi-resistant isolates were recovered from hospitalized patients during one year. Three newly isolated bacteriophages, isolated from the Parisian wastewater system, were found to lyse 42 of the 44 analyzed strains, distributed into the different clonal complexes [158].

#### 4.5.2. Nanosystems for Treatment of PACR Infections

Table 6 summarizes nanosystems to treat PACR.

Meropenem LIPs were tested against *P. aeruginosa* strains resistant to carbapenems due to low permeability or efflux, and also against isolates producing carbapenemases. Cationic LIPs were more effective against sensitive isolates compared to the free antibiotic. None of the Meropenem LIPs exhibited activity against drug-resistant isolates (due to low permeability). Resistant *P. aeruginosa* strains showed increased insusceptibility to all Meropenem LIP types [159]. Meropenem LIPs were also found more effective against *P. aeruginosa* clinical isolates compared to free drug, while they also eradicated biofilms completely and inhibited bacterial motility at lower doses [160].

Another nanosystem proposed against carbapenem-resistant bacteria are gold NPs combined with lysozyme. Lysozyme has antibacterial activity by attacking the protective wall of bacteria cells and possesses activity against Gram-positive bacteria but little against Gram-negative ones, due to the steric hindrance of the outer lipopolysaccharide layer, which blocks its access to peptidoglycans. Lysozyme-loaded NPs demonstrated enhanced antibacterial activities against imipenem-resistant *P. aeruginosa* clinical isolates, ESBL-producing *E. coli*, and a selection of Gram-negative and Gram-positive standard ATCC strains, with little or no damage to healthy human cells. This opens possible applications for wound dressings and medical devices [161].

Others evaluated the antimicrobial effects of Iron oxide NPs, alone and combined with imipenem, on *P*. *aeruginosa* isolates, producing MBLs from clinical specimens. The combined NPs inhibited *P*. *aeruginosa* growth. IONPs may negatively affect the functionality of porin pumps. Moreover, these particles may occupy the active site of MBLs, blocking their action. By impairing the antibiotic resistance mechanisms of bacteria, resistant strains became more susceptible to carbapenems [162].

### 4.6. Carbapenem-Resistant Acinetobacter Baumannii (CRAB)

#### 4.6.1. Introduction in CRAB Infections and Current Treatment Approaches

*Acinetobacter baumannii* is an opportunistic pathogen with the ability to cause severe hospital infections. It is found in many healthcare environments and is a very effective human colonizer. *A. baumannii* has emerged over the last decades as a cause of global outbreaks, displaying ever-increasing resistance rates. Reports of multi-resistant strains derive from hospitals in Europe, North and South America, Asia, and even remote areas. *A. baumannii* infections account for approximately 2% of all healthcare-associated infections in the United States and Europe [163]; however, the frequency is twice as high in Asia and the Middle East [164].

Risk factors for *A. baumannii* infections are a patient’s suppressed immune system, serious underlying diseases, invasive procedures, and treatment with broad-spectrum antibiotics, whereas among patients hospitalized in intensive care units (ICUs), they are frequently the cause of ventilator-associated pneumonia [164].

The main problem with this pathogen is the frequent occurrence of multi-resistant strains, especially in Latin America and the Middle East. According to the World Health Organization (WHO), carbapenem-resistant *A. baumannii* (CRAB) is classified in the group of critical bacteria that poses the greatest threat to human health. Carbapenems are the most potent and reliable β-lactam antibiotics for the treatment of serious infections caused by *A. baumannii. A*. *baumannii* has developed different resistance mechanisms such as carbapenemase-induced hydrolysis of the drug, changes in penicillin-binding proteins (PBPs) that prevent their affinity to the drug, alterations in the structure and number of porin proteins that result in decreased permeability to antibiotics through the outer membrane of the bacterial cell, and the activity of efflux pumps (causing resistance to different classes of antibiotics) that further decrease the concentrations of antibiotics within the bacteria [164]. Carbapenemases are enzymes capable of inactivating carbapenems. Several MBLs have been described in *A*. *baumannii*, including IMP-1, IMP-2, IMP-4, IMP-5, IMP-6, and IMP-11. These carbapenemases contribute significantly in the virulence of *A*. *baumannii* [165].

Bacteriophages with activity against *Acinetobacter* can be used as an alternative treatment. Two *A. baumannii* phages, Β/-R1215 and Β/-R2315, were isolated from sewage samples, and both were effective against almost half (21/45) of the carbapenem-resistant strains tested (isolated from respiratory samples of patients) [166]. An aerosol containing active bacteriophages was used for cleaning an intensive care unit in Taiwan. This resulted in decreased new acquisitions of CRAB infections (191 in the pre-intervention period, and 73 in the intervention period) and mean percentages of resistant strains. According to these results, phages could be used for the decontamination of hospital settings [167]. New lytic bacteriophages have also been tested in a *Galleria mellonella* and a mouse model of extensively drug-resistant *A. baumannii*-induced bacteraemia. Phages vB_AbaM_3054 and vB_AbaM_3090 were administrated alone or in combination 30 min after bacterial challenge with OXA-23 and AmpC multidrug-resistant *A. baumannii* strain FER (FER). Phage-based treatments showed high efficacy in larvae (100% survival at 80 h) and mice (100% survival at day 7) compared with the untreated controls [168].

Phages can also be used in combination with antibiotics to achieve a desirable outcome. Bacterial suppression may be stronger as a result of additive or synergistic effects. Moreover, bacteria are confronted with two different selective pressures, which prevent the emergence of resistance. Phage vB_AbaM-KARL-1, which was isolated from pond water and displays lytic activity against multi-drug-resistant clinical isolates of *A. baumannii*, was combined with meropenem, ciprofloxacin, and colistin. Phage’s activity was significantly amplified by meropenem and colistin [169].

#### 4.6.2. Nanosystems for Treatment of CRAB Infections

All nanosystems developed for CRAB infections are tabulated in Table 7.

Imipenem was conjugated on silver NPs and tested in vitro on resistant *A. baumannii* strains isolated from nosocomial infections in four hospitals in Tehran. Pathogens were isolated from urine, blood, skin, and wound, as well as from respiratory tract infections. The results revealed that among all isolated 100 clinical *A. baumannii* strains, 76% showed resistance to imipenem, with MICs ranging between 64 and 256 μg/mL. Results showed that MIC values of the conjugated with imipenem NPs were decreased, whereas low cytotoxic effects toward human fibroblasts were reported [170].

In an earlier study aiming to evaluate the effect of combined silver NPs with antibiotics to treat carbapenem-resistant *A. baumannii*, AgNP treatment showed synergistic effects with the antibiotics polymixin B and rifampicin, and an additive effect with tigecyline. In vivo, tthe combinations led to better survival ratios in *A. baumannii*-infected mouse peritonitis models compared to single drug treatment [171].

### 4.7. Neisseria Gonnorhoeae (Ng)

#### 4.7.1. Introduction in Ng Infections and Current Treatment Approaches

*Neisseria gonorrhoeae*, the aetiologic agent of gonorrhea, is the second most frequent sexually transmitted infection globally. Due to antibiotic resistance, the Center for Disease Control (CDC) listed *N. gonorrhoeae* as an urgent threat to public health. *N. gonorrhoeae* typically colonizes and infects the genital tract in men and women, but it is also found in the rectal and oropharyngeal mucosa. *Gonococcus* infects only humans in nature, and it causes urethritis in men and cervicitis in women. A minority of men (less than 10%) but a large proportion of women (>50%) can be asymptomatic. The disease is usually treated successfully with antimicrobials; however, resistance has emerged [172].

*N. gonorrhoeae* is fairly easily transmitted: the estimated probability per sex act is approximately 50% for penile-to-vaginal transmission and 20% for vaginal-to-penile transmission. If not treated properly, cervical gonorrhea can lead to severe reproductive complications, including pelvic inflammatory disease, chronic pelvic pain, ectopic pregnancies, and tubal factor infertility [173]. The WHO estimated the 2016 global prevalence of the disease at a percentage of 0.9% in women and 0.7% in men, corresponding to a total of 30.6 million cases worldwide [174].

Over time, *N. gonorrhoeae* has developed resistance to many antimicrobials including sulfonamides, penicillins, tetracycline, ciprofloxacin, and more recently, azithromycin and ceftriaxone. Penicillin resistance is associated with cumulative chromosomal mutations in different genes related with cell wall biosynthesis (*penA* and *ponA1*), or structures affecting the periplasmic drug concentration (*penB*, *penC*, and *mtrR*) [175]. Tetracycline was used as an alternative to penicillin, but resistance gradually evolved. Macrolides have also been used to treat gonorrhea, and resistance in *N. gonorrhoeae* has emerged in 2001 [176]. The combination scheme introduced in the US and Europe appears to be currently highly effective. However, the susceptibility to ceftriaxone has decreased in recent years, and resistance to azithromycin is prevalent in many settings, especially where frequently used. Subsequently, gonococcal strains with decreased susceptibility or resistance to ceftriaxone /azithromycin are already circulating globally.

#### 4.7.2. Nanosystems for Treatment of Ng Infections

As seen in Table 8, LIPs have been used as nanosystems to treat Ng infections in some cases, as described below. Mupirocin cannot be used against gonococcus due to its rapid systemic elimination and high protein binding. Mupirocin LIPs enabled its efficacy after injection in mice; thereby, high in vivo anti-bacterial activity against resistant *N. gonorrhoeae* strains was possible [177]. In another study, LIP octylglycerol (OG) was developed as a vaginal microbicide. The formulation demonstrated in vitro activity against several pathogens, including *Neisseria gonorrhoeae* and HSV-1, HSV-2, and HIV-1. Toxicity of OG-LIP toward *Lactobacillus* was avoided by controlling the OG/lipid ratio. Toxicity was assessed ex vivo on excised human tissues and in vivo in macaques (rectal administration). LIP had no toxicity toward ectocervical tissues and no effect on the rectal pH and microbiological flora, while they also did not cause epithelial desquamation [178]. LIPs were also used for the development of a multiplexed immunoassay chip with five antibodies against pathogens that can cause female lower genital tract infections (*N. gonorrhoeae*, *Chlamydia trachomatis*, *E. coli*, *Streptococcus agalactiae*, *Candida albicans*). The test was inexpensive, quick (results in 3 h), and with high specificity/sensitivity to detect *Candida* infection [179].

In another case, inactivated whole cells of gonococci strain CDC-F62 were spray-dried and encapsulated into a biodegradable cross-linked albumin matrix, resulting in sustained and slow antigen release. The particles were loaded in microneedles for transdermal administration, as a vaccine antigen, and assessed in vitro (on dendritic cells and macrophages) and in vivo in mice. Increases in antigen-specific IgG antibody titers and antigen-specific CD4 and CD8 T lymphocytes in mice were observed compared to free gonococcal antigens or empty microneedles. The transdermal vaccine delivery promoted immune system responses to bacterial antigens, since various immune cells are accessible. The microparticle-in 0-microneedle-patch was effective for gonococci treatment/prevention [180].

Silver NPs with cefmetazole were also used, and they demonstrated lower MIC values than the antibiotic alone. Cefmetazole NPs delivered over twice the antibiotic dose to bacteria, and hindered drug-resistant mechanisms of *N. gonorrhoeae*, opening the way for applications such as topical treatments or coatings for biomaterials [181].

### 4.8. Mycobacterium Tuberculosis (MTB)

#### 4.8.1. Introduction in MTB Infections and Current Treatment Approaches

*Mycobacterium tuberculosis* (MTB) is a strictly aerobic, branched, or rod-shaped intracellular bacterium that causes tuberculosis (TB). Today, TB has infected (in many cases asymptomatically) more than two billion people worldwide, being thus considered as a global public health problem. In 2019, 8.9–11.0 million new TB cases and close to 1.6 million deaths were reported [182].

Immotile by nature, MTB can only invade skin and mucous membranes where integrity is compromised. Infections are mostly systemic, and they may invade both the lungs and extrapulmonary organs, such as bones, kidneys, ovaries, meninges, and others. Due to its ability to be aerosolized, MTB can spread (by air-borne droplets or dried sputum) through the upper respiratory tract (by coughing or sneezing). Patients with active TB can transmit MTB to healthy people [182,183].

Treatment success rates highly vary, ranging from 28% (for extensively drug-resistant strains) to 83% (for susceptible strains). The rate of decline in TB incidence is almost stagnant at 1.5% annually over the past decade, which is behind the projected by the WHO’s End TB Strategy of 5%. TB remains among the top 10 causes of death worldwide [182], with the WHO estimating that up to 1/3 of the world’s population is infected (actively or latently) by the pathogen [182,183]. HIV co-infections and senescence, as well as co-morbidities such as diabetes, are among the risk factors for active disease development. In addition, weakened immune systems are highly susceptible to active disease, which is a fact exacerbated by the rapidly aging populations [183].

*M. tuberculosis* is an intracellular bacterium that after phagocytosis by macrophages can survive and multiply in the host cell. The development of an adaptive immune response mediated by T-lymphocytes (by the production of macrophage-activating cytokine interferon-gamma (IFN-γ), macrophages, and lymphocytes toward the formation of granulomata, are crucial for the control of *M. tuberculosis* [7,184].

First-line anti-TB treatment (isoniazid, rifampicin, ethambutol, and pyrazinamide) is often used initially, and second-line drugs (kanamycin, amikacin, fluoroquinolones, and capreomycin) are used in cases of resistance and/or treatment failure. Conventional TB therapy includes daily treatments with high doses of antimycobacterial drugs for at least 6 months. Such regimens come with severe side effects and poor compliance, which is one of the main reasons for multidrug-resistant (MDR) strain emergence, especially in developing countries. Thus, improvement of antimycobacterial therapy is an urgent need. The development of new drugs is an obvious strategy, but mechanisms to improve the efficacy of existing drugs may be much faster. Efficacy improvement can be achieved by increasing drug concentrations at infected sites or by reducing toxic effects and/or the duration of treatments. The latter improvements are possible by developing nanosystems for drug administration [185].

Bacteriophages are also being recently revisited as antimicrobial treatments of MTB infections. In this context, anti-tuberculosis bacteriophage D29 was found to protect against *M. tuberculosis* infection, since pre-treatment with bacteriophage aerosol significantly decreased *M. tuberculosis* burden in mouse lungs [186]. Futhermore, three inhalation devices were compared for their potential to deliver active anti-tuberculosis bacteriophage D29 to the lungs [187], while others evaluated atmospheric spray freeze-drying (ASFD) as a method to formulate bacteriophage D29 into a dry solid [188].

#### 4.8.2. Nanosystems for Treatment of MTB Infections

As LIPs passively target macrophages, the main hosts of bacteria in MTB infections, they have many applications for MTB treatment, as summarized in Table 9.

Conventional and actively targeted and/or stimuli-responsive LIPs can be used, the latter to further increase drug accumulation in specific tissues and/or cells [189].

Several reports test LIP formulations toward *M. tuberculosis* strains in vitro. Different LIP types were loaded or co-loaded with artemisone, clofazimine, and decoquinate anf tested toward the *M. tuberculosis* H37Rv strain; the highest percentage inhibition (52%) was obtained with niosomes containing 1% clofazimine [190]. Cardiolipin-containg LIPs completely suppressed *M. tuberculosis*, while levofloxacin LIPs (PC/Chol/cardiolipin) further reduced the MIC of cardiolipin LIPs and the drug [191]. An improved intracellular uptake of usnic acid (UA) by J774 macrophages and prolonged retention in the cells was demonstrated from UA LIPs compared to free UA [192]. Anionic LIPs loaded with UA had dramatically decreased MIC compared to UA. Co-entapped RIF and UA exhibited a synergistic interaction against MDR-TB clinical isolates, while synergist effects were not seen with INH [193]. Zn(II)-phthalocyanine LIPs showed photodynamic activity against two strains of *M. tuberculosis*: a susceptible (ATCC 27294) and an MDR one [194].

The oxadiazole derivative (palmitic acid conjugate) of isoniazid (INH) has anti-mycobacterial activity against *M. tuberculosis* strains, and when loaded in LIPs consisting of PC (phosphatidylcholine)/PA (L-a phosphatidic acid), or PC/Chol, it exhibited high anti-mycobacterial activity in MDR *M. tuberculosis* infected BALB/c mice [195].

Rifampicin (RIF) was loaded in aerosolized LIPs to target alveolar macrophages, which is the most important site for MTB infection. PC/Chol LIPs were modified by (i) the incorporation of dicetylphosphate (DCP) in their membranes for negative charge, or (ii) by surface coating with macrophage binding ligands, such as maleimide bovine serum albumin (MBSA) and O-steroyl amylopectin, (O-SAP). The coated-LIPs accumulated highly in lung macrophages (compared to non-coated ones). Higher sustained RIF amounts were found in lungs receiving modified LIPs compared to free drug and plain LIPs. RIF in lungs 6 h post-administration of targeted-LIPs (with MBSA or O-SAP) was 1.4 and 3.5 fold higher than those acheived with negative charge and plain LIPs, respectively, indicating the superiority of the coated-LIPs [184].

Stealth MLV-LIPs that consisted of PC/Chol/DCP/DSPE-PEG were conjugated with O-stearylamylopectin (O-SAP) for lung targeting. The co-encapsulation of INH and RIF in the LIPs at 33% of their recommended doses exhibited sustained levels of both drugs in plasma, lungs, liver, and spleen, conferring higher bioavailabilities compared to free drug administration. Administration of one dose of LIPs per week, for 6 weeks, resulted in a significant reduction of mycobacterial loads compared to untreated mice [196].

Rifabutin (RFB) is used for treatment of infections by *M. tuberculosis*, *Mycobacterium leprae*, and *M. avium*. RFB-LIPs conferred higher drug levels in liver, spleen, and lungs, compared to free RFB. RFB levels in organs were dependent on LIP composition; LIPs consisted of dipalmitoylphosphatidylcholine and dipalmitoyl phosphatidyl glycerol (DPPC/DPPG) being most effective and achieving lower bacterial loads in spleen and liver in vivo. Treatment with RFB-LIPs is a promising approach for extrapulmonary TB in patients that are co-infected with HIV [197]. Dextrazide (DZ), a dextran conjugate of isonicotinic acid hydrazide (INAH), shows lysosome tropism and prolongs the intracellular activity of INAH. DZ-LIPs, which consisted of PC, were tested in a mouse model of MBT; DZ-LIPs alone and DZ-LIPs+INAH demonstrated highest efficacy against cell persistent circulating MBT. The LIPs have lower hepatotoxicity (compared to free drug) and are promising for acute lung TB treatment [198]. Pyrazinamide (PZA) was successfully encaspsulated in LIPs, exhibiting high therapeutic efficacy against *M. tuberculosis* in mice. PZA-loaded neutral LIPs (DPPC/Chol) and negatively charged LIPs (DPPC/Chol/DCP) could confer a significant reduction in bacterial counts even 30 days after the last treatment, while a histopathological examination of lungs showed the highest severity of infection in drug-free LIP group (control) compared with PZA-LIP treated mice [199]. Amikacin-LIPs, when administered by 3 injections/week, were found to be 2.7–2.9 times more active than free amikacin and 3.7–5.6 more active than streptomycin. In a model of chronic TB, treatment with amikacin LIPs (3/week for 4 weeks and then 1/week for 4 weeks) had greater bactericidal activity than free amikacin (5/week) but lower sterilizing activity than oral INH or RIF (5/week) [200].

First-line anti-tubercular drugs (ATDs), i.e., RIF, INH, and PZA were also tested as polymeric NP formulations. Recently, poly (DL-lactide-coglycolide) NPs (PLG-NPs) were explored for their suitability as antitubercular drug carriers for oral/aerosol administration. Dosing frequency could be reduced to once/10 d, instead of daily with conventional therapy. PLG-NPs encapsulating the three ATDs were prepared by multiple emulsion, and one dose of PLG-NPs could sustain the therapeutic plasma levels for 32 d and for 36 d in lungs and spleen, respectively. No bacterial counts were found in the lungs and spleen of MBT-infected mice, demonstrating superior chemotherapeutic efficacy compared with daily dosing of the free drugs [201]. ATD-loaded PLG-NPs realized detectable drug levels in blood for 6 d (RIF) or even 9 d (INH/PZA) after one oral administration, while therapeutic tissue levels were maintained for 9–11 days. In MBT-infected mice treated once every 10 days, tubercle bacilli could not be detected at all after five treatments [202]. Wheat-germ agglutinin (lectin) (WGA)-coated and ATD-loaded PLG-NPs administered to guinea pigs through the oral/aerosol route confered detectable drug levels in plasma for 6–7 days for RIF and 13–14 days for INH/ PZA. All drugs were present for 15 days in lungs, liver, and spleen. Efficacy studies revealed that three doses of NPs (administered with 2-week intervals), diminished lung and spleen mycobacterial counts (cfu), when 45 doses of free drugs were required for the same effect [203]. In another case, nebulization of ATD-loaded PLG-NPs was found to improve drug bioavailability and reduce dosing frequency for the treatment of pulmonary tuberculosis (in guinea pigs). A single nebulized dose realized therapeutic plasma levels for 6–8 days and for 11 days in lungs. No tubercle bacilli were found in lungs of *tuberculosis* infected guinea pigs after five doses of nebulized NPs (administered at 10-day intervals); 46 daily oral doses of free drug were required for equivalent results [204].

In another case, RIF was formulated into powders for aerosol delivery in porous particles without NPs and particles with PLGA-NPs (PNAPs). PNAPs conferred systemic RIF levels for 6-8 h post-intratracheal insufflation to guinea pigs, and detectable levels in lung for >10 h, which could not be achieved by powders without NPs, proving the potential of the NP formulation for enhanced systemic exposure of RIF in the lungs [205].

Oral delivery of ethionamide (an effective widely used second-line drug for MDR TB)-loaded in PLGA-NPs realized therapeutic drug plasma levels for 6 days compared to 6 h after delivery of the free drug. Organ (lung, liver, and spleen) drug levels were detectable for up to 5–7 days after NP delivery compared to 12 h with the free drug. The PLGA-NPs improved drug pharmacodynamics and pharmacokinetics, indicating their potential to reduce ethionamide dosing frequency and related GI intolerance, for efficient/safe treatment of MDR TB [206].

As mentioned before, Bacteriocin (Bcn) peptides are secreted by bacteria and exert bactericidal activity against other bacterial species. Bcn5 (selected as a model compound due to its cytotoxicity and antimycobacterial activity) was loaded in PC/cardiolipin LIPs and demonstrated ability to inhibit intracellular growth of virulent *M. tuberculosis* H37Rv and prolong the survival of mice with acute TB [207].

Recent cases of non-lipidic NPs studied in vitro against MTB include graphene oxide (GO) nanocarriers co-loaded with Rifampicin and Isoniazid and coated with chitosan and Gum Tragacanth [208]; Rifampicin and curcumin co-loaded polymeric NPs that showed high efficacy against MTB infected macrophages [209]; Rifabutin-loaded glucan microparticles that demonstrated enhanced protection against intracellular *M. tuberculosis* [210], and Moxifloxacin poly(butyl cyanoacrylate (PBCA) NPs that improved the drug effect against *M. tuberculosis* in macrophages [211].

### 4.9. Mycobacteria Other Than Tuberculosis (MOTT)

#### 4.9.1. Introduction in MOTT Infections and Current Treatment Approaches

Mycobacteria other than tuberculosis (MOTT) are widespread environmental bacteria that cause infections at various anatomical sites, mostly in people with impaired immunity. Due to their hydrophobicity, MOTT species are abundant in different environments such as water, soil, foods, etc., can survive at high temperatures and in low pH media, and can be aerosolized and form biofilms. The molecular mechanisms of MOTT-induced infections are similar to that of tuberculosis [212,213]. Inhalation of MOTT-contaminated aerosolized droplets is the mode of transmission of pulmonary infections [213].

The most common infection caused by MOTT is lung disease, which is a chronic life-threatening condition usually caused by *Mycobacterium avium* complex (MAC) (*M. avium* and *Mycobacterium intracellulare*). MOTT infections can be lethal for immunocompromised patients such as HIV and patients that received transplants. Although MOTT-induced infections are generally not contagious, recent evidence suggests transfer among cystic fibrosis patients. MOTT lung disease prevalence has been increasing in the last few years [213]. Treatment of MOTT lung disease is challenging; regimens containing a macrolide, ethambutol, and a rifamycin (rifampin or rifabutin) are usually considered. Intravenous aminoglycosides (streptomycin or amikacin) can be added to the regimen, but their use is limited due to toxicity [213].

#### 4.9.2. Nanosystems for MOTT Infection Treatment

All of the advanced reports (that include in vivo studies) about nanosystems to treat MOTT infections involve LIPs. In this context, several drugs have been tested in LIP form, such as rifabutin, ciprofloxacin, and amikacin. Rifabutin LIPs composed of PC and phosphatidylserine were tested in a virulent *M. avium* strain (strain P1581) infected murine model, and significant efficacy enhancement was observed (compared to free drug) in therapeutic and prophylactic treatment protocols [214]. More recently, ciprofloxacin LIPs were developed for the treatment of tuberculosis and MOTT infections caused by MAC in patients with AIDS. LIPs were significantly more active than free drug against macrophage- and biofilm-located pathogens. In vivo, ciprofloxacin LIPs significantly decreased bacterial loads in lungs of mice infected with *M. avium* and *M. abscessus*, suggesting that topical LIP delivery can be an effective treatment of MOTT [215].

Most of the effort for nanosystems to treat MOTT infections has been dedicated up to now, to studies evaluating the potential of amikacin-loaded liposomes. These studies finally resulted in the development of a product. ALIS; Arikayce^®^, or LAI, is an amikacin LIP inhalable suspension for local delivery to the lungs (for limited systemic exposure) by inhalation after nebulization [216].

Importantly, in the FDA site announcing approval of Arikayce [217], it is stated that “Arikayce is the first drug to be approved under the ‘Limited Population Pathway for Antibacterial and Antifungal Drugs’ or LPAD pathway, established by Congress under the 21st Century Cures Act to advance development and approval of antibacterial and antifungal drugs to treat serious or life-threatening infections in a limited population of patients with unmet needs. Approval under the LPAD pathway may be supported by a streamlined clinical development program. These programs may involve smaller, shorter, or fewer clinical trials. As required for drugs approved under the LPAD pathway, labeling for Arikayce includes certain statements to convey that the drug has been shown to be safe and effective only for use in a limited population”.

ALIS was FDA approved “as part of a combination antibacterial drug regimen for MAC lung disease treatment in adult patients who have not achieved negative sputum cultures despite ≥6 consecutive months of a multidrug background regimen therapy and who have limited or no alternative treatment options” [217].

Previous studies have shown that ALIS LIPs efficiently penetrate *M. avium* biofilms in vitro and reduce the viable cell counts in a dose-responsive way; they also effectively reduce the cell counts of *M. avium* and *M. abscessus* in infected macrophages, and they were more effective against intracellular *M. avium* and *M. abscessus* compared to free amikacin. Applied against *M. avium* in a mouse model of respiratory infection, the formulations induced a significant reduction in the mouse *M. avium* load, which was comparable to the reduction conferred by parenterally administered free amikacin. ALIS also realized rapid and complete elimination of the *mycobacteria* in all infected organs within 12 weeks of treatment without relapse of infection, while treatment duration could be reduced to 12 weeks (as compared to 24 weeks of daily treatment with free clarithromycin (6 days a week)). As proven by clinical studies, the addition of ALIS to guideline-based therapy (GBT) for the treatment of refractory MOTT lung diseases resulted in higher response rates in microbiological endpoints compared to GBT alone [218,219,220,221,222].

## 5. Clinical Studies, Products, and Future Perspectives

**Figure 4:**
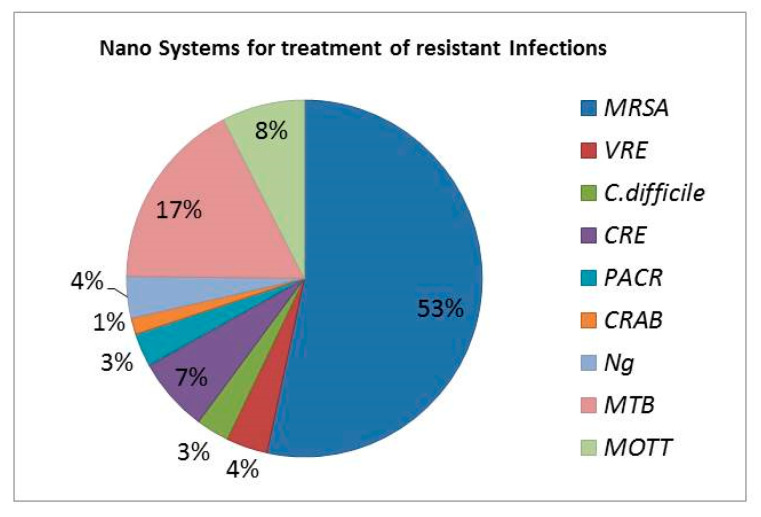
Distribution of nanosystems reported for resistant infection treatment per pathogen.

Summarizing the previous section, it is evident that numerous lipidic and non-lipidic nanosystems containing antimicrobials or antimicrobial combinations have been developed and evaluated in vitro and in vivo in the last 20 years (in total 133), with the final aim being to treat resistant microbial diseases. From the nine pathogens reviewed, most efforts involve *MRSA* (53%) and *Mycobacterium tuberculosis* and MOTT (cumulatively 25%) infections (Figure 4).

Between the 133 nanosystems reported, 81 (≈61%) involve liposomes (mostly) and lipidic NPs and 52 (≈39%) involve non-lipidic NPs, many of which are inorganic NPs loaded or associated with drugs. It should be reminded at this point that reports involving plain metallic/inorganic NPs (having intrinsic antibacterial properties) not in combination with drugs have not been included in this review.

Considering the application of bacteriophages for the treatment of infections, recent studies have been reported for almost all pathogens investigated (37 reports in total); however, the cases where bacteriophages were introduced/or associated with nanosystems for their delivery are limited (only 2). Nevertheless, the results of these first approaches are extremely promising, indicating the high potential of this strategy, which is certainly worthy of further future exploitation. Indeed, overcoming the challenge to develop optimal pharmaceutical products for bacteriophage delivery, which would be able to preserve their activity, would probably realize a great breakthrough for the treatment of persistent and resistant infections.

Regarding the current approved nanosystem products, as already mentioned above, an LIP product, Arikayce^®^ has been approved by FDA in 2018 as part of a combination antibacterial drug regimen for MAC lung disease treatment under specific conditions [217].

In regard to studies in clinical settings, searches in the clinical study database [223] using the keywords: “Bacterial” AND “Infection” led to one hit when the word “nanoparticle” was added and 12 hits when the word “liposome” was used instead (see Table S1 in Supplementary Materials). From the twelve investigations about liposomal formulations (at various status: completed, recruiting, or unknown), most (7) are studies involving liposomal amikacin for inhalation, to treat various conditions, i.e., *Mycobacterium nontuberculous* infections (4), bronchiectasis (1), cystic fibrosis (1) and chronic *Pseudomonas aeruginosa* infections. From the remaining clinical studies with liposomes, two involve liposomal amphotericin B to treat cryptococcal meningitis; two more involve a liposomal product of cytarabin (DepoCyt^®^) to treat neoplastic meningitis; and finally, one study involves a new liposomal adjuvant given with the tuberculosis subunit vaccine. The one study involving NPs concerns the topical application of silver NPs to treat foot infections. From the previous, is becomes evident that all of the studies reported in paragraph 4 involving nanosystems for treatment of the resistant pathogens considered herein (Figure 3), with the exception of amikacin liposomes, which have not yet advanced to the level of clinical testing.

Nevertheless, from the various study results presented herein, it is clear that the potential of nanosystems for delivery and/or targeting (in some cases) of different types of antimicrobial substances (such as drugs, peptides, bacteriophages, inorganic NPs) or their combinations, into infected sites and cell types, is indeed a very promising strategy for the treatment of persistent and drug-resistant infections.

Table 2, Table 3, Table 4, Table 5, Table 6, Table 7, Table 8 and Table 9 summarize the different strategies and approaches considered to date, involving the development of nanosystems for the treatment of drug-resistant infections and their possible strategies to achieve increased therapeutic efficacy. We may conclude that in addition to the sustained delivery and modulation of drug distribution/disposition that confer therapeutic advantages by increasing the efficacy and/or decreasing the toxicity of drugs, various “smart” nanosystems have been designed in order to deliver high amounts of drug to target infected sites by (i) using special types of NPs (such as elastic LIPs or transferosomes for skin delivery, or inhalable LIPs for lung infections), (ii) by enhancing the release of drug in the presence of specific environment or pathogen-related triggers, (iii) by actively targeting specific bacterial components, or (iv) by increasing with different approaches the penetration of drugs to target cells even within persistent biofilms. Additionally, improved efficacy has been also achieved by the strategy of combining substances with different mechanisms of action in the same NP in order to confer synergistic results. Another strategy for the augmentation of antibacterial activity of drugs is their conjugation with inorganic NPs that have intrinsic antimicrobial properties. It is also important to state that in most cases, the toxicities of the systems proposed are low or negligible.

Concluding, we anticipate that the search for optimal nanosystems toward the eradication of persistent and MDR pathogens will continue in the near future, hopefully resulting in the development of successful therapeutic systems to overcome the currently unmet medical needs in the area of infectious diseases.

## Figures and Tables

**Table 1:** Antibacterial agents and their targets in bacteria.

Bacterial Target	Groups	Agents
**Cell wall synthesis**	β-lactams	Penicillins, cephalosporins, cabapenems, monobactams, beta-lactamase inhibitors
	Glycopeptides, Lipoglycopeptides	Vancomycin, teicoplanin, dalbavancin, telavancin
**Cell membranes’ damage**	Polymyxins, Lipopeptides	Colistin, daptomycin
**Inhibitors of protein synthesis**	Targeting 50SrRNA	Macrolides, lincosamides, streptogramins, chloramphenicol, oxazolidinones
	Targeting 30SrRNA	Aminoglycosides, tetracyclines
**Inhibitors of nucleic acids’ synthesis**	Targeting RNA	Rifamycins
	Targeting DNA	Quinolones, metronidazole, nitrofurans
**Antimetabolites**	Inhibition of folic acid synthesis	Trimethoprim, sulfamethoxazole

**Table 2:** Nanosystems for treatment of MRSA infections.

NS Type	Strategy/Observations/Results	Ref.
**Phages (free)**	Phage K, Phages +drugs → synergism against biofilm (also in vivo)	[29,30,31,32,33,34,35]
**LIPs**	↑ drug deposited in infected tissues; Controlled release;	[39,41,50,52,56,60,61,62,63,64,65,66,67,70,71,72,73]
**PEG-LIPs**	Prolonged circulation/modified pharmacokinetics (↑ activity and/or ↓ toxicity); Synergistic/additive effects of drug combinations;	[38,42,43,53,75,76,77,81,82]
**Targeted LIPs**	Specific administration (inhalable, skin); Bacterial (toxin) triggered; Fusogenic (penetrate biofilms); Ligand-targeted; pH-sensitive;	[40,43,44,45,46,48,49,57]
**Special LIPs**	Elastic; Bacterial toxin specific; Polycationic (disorganize bacterial wall and ↑ permeability); Anionic or Cationic for ↓ of genes;	[54,68,69,74,79,80]
**Hybrid-LIP**	Polymer-embedded/coated, for optimal encapsulation, release, disposition;	[47,51,55,58]
**Phage-LIPs**	Phage cocktails → ↑ Phage persistence; Effective Phage delivery;	[15,78]
**Inorganic-NPs**	Metallic-NPs + drugs → additive or synergistic effects	[84,85,86,87,88,89,90,91,93,94,95,96,97]
**Polymer-NPs**	Modified drug disposition & release; ↑ activity of water insoluble drugs; ↑ enzymatic stability;	[98,99,100,101,102,103,104,106]
**Targeted Non-lipid NPs**	NPs bind to MRSA surface → ↑ efficacy; ↑ cellular uptake of drugs; ↑ drug release in presence of bacterial lipases;	[92,105,107]

**Table 3:** Nanosystems for the treatment of VRE infections.

NS Type	Strategy/Observations/Results	Ref.
**Phages (free)**	SHEF2, Phage cocktails etc. effective also for biofilm forming strains	[14,18,111]
**LIPs**	Fatty acids and cholesteryl esters in LIPs → ↑ activity	[116]
**Inorganic-NPs**	Metallic-NPs + drug → effective in dose dependent manner; ↓ MIC; enhanced activity;	[113,115]
**Polymer-NPs**	Effective to remove biofilms of MDR bacteria; NPs in bacteria biofilms → ↑ dispersal;	[117]
**Targeted Non-lipid NPs**	Magnetic NPs with pathogen receptor-specific ligand → polyvalent effects, pathogen detection	[114]

**Table 4:** Nanosystems for treatment of CDI infections.

NS Type	Strategy/Observations/Results	Ref.
**Phages (free)**	Different phages → Alter bact. toxin production; combinations → ↓ colonization;	[118,122,123,124,125]
**LIPs**	Curcumin LIPs → efficacy not improved;	[131]
**Special LIPs**	Cationic bile-acdi LIPs for oligo delivery → nM MICs for 4 gene targets; Elastic; Bacterial toxin specific; Polycationic (disorganize bacterial wall and ↑ permeability); Anionic or Cationic for ↓ of genes;	[130]
**Inorganic-NPs**	Metallic-NPs + drug → specific binding to CD spores and ↑ efficacy	[128]
**Polymer-NPs**	High drug load and mucoahvesive pror.; Modified drug disposition & release; ↑ activity of water insoluble drugs; ↑ enzymatic stability;	[129]

**Table 5:** Nanosystems for treatment of CRE infections.

NS Type	Strategy/Observations/Results	Ref.
**Phages (free)**	Phage + solar irrad. → ↑ bacteria susceptibility; Phage cocktails → ↑ activity against resistant & biofilm producing bacteria;	[138,139,140,141,142]
**Lipid-NPs**	SLN oligonucleotides → in vitro efficacy; SLN + drug → prolonged antibacterial activity;	[143,144]
**Inorganic-NPs**	Metallic-NPs + drug, synergism & ↑efficacy in MDR infections; ↑ efficacy (in vivo); ↓ MICs;	[145,146,147,148,149]
**Polymer-NPs**	↑ activity against MDR infection & survival, ↑bacterial clearance in vivo; controlled release; biocompatible;	[150,151,152]

**Table 6:** Nanosystems for treatment of PACR infections.

NS Type	Strategy/Observations/Results	Ref.
**Phages (free)**	Phage OA392 & others → effective in PACR infections;	[156,157,158]
**LIPs**	Cationic-LIP → ↑ efficacy against sensitive isolates than free drug; Resistant bacteria had ↑ insusceptibility for all LIP-types	[159,160]
**Inorganic-NPs**	Metallic-NPs + drugs → inhibited bacterial growth and ↑activity against resistant isolates; no harm to human cells; acting by blocking the action of bacterial- produced MBLs;	[161,162]

**Table 7:** Nanosystems for treatment of CRAB infections.

NS Type	Strategy/Observations/Results	Ref.
**Phages (free)**	B/-R1215 & B/-R2315, active against resistant strains; Aerosol phage as disinfectant for IC units; Novel phages & cocktails → ↑ in vivo efficacy; Combinations with antibiotics → strong synergism;	[166,167,168,169]
**Inorganic-NPs**	Metallic-NPs + drugs → ↓ MICs of drugs; synergistic or additive effects with antibiotics; ↑survival (in vivo);	[170,171]

**Table 8:** Nanosystems for treatment of Ng infections.

NS Type	Strategy/Observations/Results	Ref.
**LIPs**	LIP enabled drug efficacy by prolonging systemic retention → ↑ efficacy; ↑ efficacy of LIP microbicide (in vitro) and good biocompatibility; LIP applied for pathogen detection chip;	[177,178,179]
**Non-Lipid NPs**	Gonococci strain antigens in spray-dried albumin matrix → ↑ immuno-response compared to free antigen;	[180]
**Inorganic-NPs**	Metallic-NPs + drug → ↓ MICs of drug; hindered drug resistance;	[181]

**Table 9:** Nanosystems for treatment of MTB infections.

NS Type	Strategy/Observations/Results	Ref.
**Phages (free)**	Phage D29 → protective effect; inhalation devices and spray drying of D29 evaluated;	[186,187,188]
**LIPs**	LIP target macrophages → pathogen site; Inhibition or complete suppression of *M. tuberculosis*; with different drug-loaded LIP (depending on lipid comp.); ↑ intracellular drug uptake & retention; Synergistic/additive effects of drug combinations; lower drug toxicity; Bacteriocin-LIPs → ↓ pathogen growth & prolonged survival;	[189,190,191,192,193,194,195,197,198,199,200,207]
**PEG-LIPs**	Stealth & lung targeted-LIP with drug combination → more drugs at site → ↑ Efficacy;	[196]
**Targeted LIPs**	Aerolised LIPs& target to alveolar macrophages → Higher sustained drug amounts in lungs with targ.LIP;	[184]
**Polymer-NPs & targeted-PNPs**	Reduced dosing frequency and ↑ bioavailability with NPs (even more when nebulized); ↑ and prolonged activity; Lectin-Targeted NPs → diminished bacteria in macrophages; Drug combinations → ↑ efficacy; Oral delivery of NPs showed efficacy in vivo;	[201,202,203,204,205,208,209,210,211]

## Data Availability

Not applicable.

## Supplementary Materials

The following are available online at https://www.mdpi.com/article/10.3390/nano11051075/s1, Table S1: Clinical Studies involving nanosystems to treat bacterial infections.

## References

[1] https://pubmed.ncbi.nlm.nih.gov.

[2] Yetisgin A.A., Cetinel S., Zuvin M., Kosar A., Kutlu O. (2020). Therapeutic nanoparticles and their targeted delivery applications. Molecules.

[3] Dawar S., Singh N., Kanwar R.K., Duan W., Kanwar J.R. (2013). Multifunctional and multitargeted nanoparticles for drug delivery to overcome barriers of drug resistance in human cancers. Drug Discov. Today.

[4] Antimisiaris S.G., Kallinteri P., Fatouros D.G., Gad S.C. (2008). Liposomes and drug delivery. Pharmaceutical Manufacturing Hanbook: Production and Processes.

[5] Antimisiaris S.G., Marazioti A., Kannavou M., Natsaridis E., Gkartziou F., Kogkos G., Mourtas S. (2021). Overcoming barriers by local drug delivery with liposomes. Adv. Drug Deliv. Rev..

[6] Bankier C., Matharu R.K., Cheong Y.K., Ren G.G., Cloutman-Green E., Ciric L. (2019). Synergistic antibacterial effects of metallic nanoparticle combinations. Sci Rep..

[7] Lee N.-Y., Ko W.-C., Hsueh P.-R. (2019). Nanoparticles in the treatment of infections caused by multidrug-resistant organisms. Front. Pharmacol..

[8] Levinson W., Chin-Hong P., Joyce E.A., Nussbaum J., Schwartz B., Hill M. (2020). Review of Medical Microbiology & Immunology: A Guide to Clinical Infectious Diseases.

[9] Lin D.M., Koskella B., Lin H.C. (2017). Phage therapy: An alternative to antibiotics in the age of multi-drug resistance. World J. Gastrointest. Pharmacol. Ther..

[10] Yilmaz C., Colak M., Yilmaz B.C., Ersoz G., Kutateladze M., Gozlugol M. (2013). Bacteriophage therapy in implant-related infections: An experimental study. J. Bone Jt. Surg. Am..

[11] Gutiérrez D., Fernández L., Rodríguez A., García P. (2018). Are phage lytic proteins the secret weapon to kill *Staphylococcus aureus*?. mBio.

[12] Fischetti V.A. (2018). Development of phage lysins as novel therapeutics: A historical perspective. Viruses.

[13] Kurlenda J., Grinholc M. (2012). Alternative therapies in *Staphylococcus aureus* diseases. Acta Biochim. Pol..

[14] Barros J., Melo L.D.R., Poeta P., Igrejas G., Ferraz M.P., Azeredo J., Monteiro F.J. (2019). Lytic bacteriophages against multidrug-resistant *Staphylococcus aureus*, enterococcus faecalis and escherichia coli isolates from orthopaedic implant-associated infections. Int. J. Antimicrob. Agents.

[15] Chhibber S., Shukla A., Kaur S. (2017). Transfersomal phage cocktail is an effective treatment against methicillin-resistant *Staphylococcus aureus*-mediated skin and soft tissue infections. Antimicrob. Agents Chemother..

[16] Alves D.R., Gaudion A., Bean J.E., Esteban P.P., Arnot T.C., Harper D.R., Kot W., Hansen L.H., Enright M.C., Jenkins A.T.A. (2014). Combined use of bacteriophage K and a novel bacteriophage to reduce *Staphylococcus aureus* biofilm formation. Appl. Environ. Microbiol..

[17] Pincus N.B., Reckhow J.D., Saleem D., Jammeh M.L., Datta S.K., Myles I.A. (2015). Strain specific phage treatment for *Staphylococcus aureus* infection is influenced by host immunity and site of infection. PLoS ONE.

[18] Gelman D., Beyth S., Lerer V., Adler K., Poradosu-Cohen R., Coppenhagen-Glazer S., Hazan R. (2018). Combined bacteriophages and antibiotics as an efficient therapy against VRE *Enterococcus faecalis* in a mouse model. Res. Microbiol..

[19] Mattila S., Ruotsalainen P., Jalasvuori M. (2015). On-demand isolation of bacteriophages against drug-resistant bacteria for personalized phage therapy. Front. Microbiol..

[20] Hua Y., Luo T., Yang Y., Dong D., Wang R., Wang Y., Xu M., Guo X., Hu F., He P. (2017). Phage therapy as a promising new treatment for lung infection caused by carbapenem-resistant *Acinetobacter baumannii* in mice. Front. Microbiol..

[21] Bernasconi O.J., Donà V., Tinguely R., Endimiani A. (2017). In Vitro activity of three commercial bacteriophage cocktails against multidrug-resistant *Escherichia coli* and proteus spp. strains of human and non-human origin. J. Glob. Antimicrob. Resist..

[22] Elbreki M., Ross R.P., Hill C., O’Mahony J., McAuliffe O., Coffey A. Bacteriophages and Their Derivatives as Biotherapeutic Agents in Disease Prevention and Treatment. https://www.hindawi.com/journals/jvi/2014/382539/.

[23] Poovelikunnel T., Gethin G., Humphreys H. (2015). Mupirocin resistance: Clinical implications and potential alternatives for the eradication of MRSA. J. Antimicrob. Chemother..

[24] Gilmer D.B., Schmitz J.E., Euler C.W., Fischetti V.A. (2013). Novel bacteriophage lysin with broad lytic activity protects against mixed infection by streptococcus pyogenes and methicillin-resistant *Staphylococcus aureus*. Antimicrob. Agents Chemother..

[25] Plotka M., Kapusta M., Dorawa S., Kaczorowska A.-K., Kaczorowski T. (2019). Ts2631 endolysin from the extremophilic thermus scotoductus bacteriophage VB_Tsc2631 as an antimicrobial agent against gram-negative multidrug-resistant bacteria. Viruses.

[26] van Nood E., Vrieze A., Nieuwdorp M., Fuentes S., Zoetendal E.G., de Vos W.M., Visser C.E., Kuijper E.J., Bartelsman J.F.W.M., Tijssen J.G.P. (2013). Duodenal infusion of donor feces for recurrent *Clostridium difficile*. N. Engl. J. Med..

[27] Tong S.Y.C., Davis J.S., Eichenberger E., Holland T.L., Fowler V.G. (2015). *Staphylococcus aureus* infections: Epidemiology, pathophysiology, clinical manifestations, and management. Clin. Microbiol. Rev..

[28] Vestergaard M., Frees D., Ingmer H. (2019). Antibiotic resistance and the MRSA problem. Microbiol. Spectr..

[29] Mann N.H. (2008). The potential of phages to prevent MRSA infections. Res. Microbiol..

[30] O’Flaherty S., Ross R.P., Meaney W., Fitzgerald G.F., Elbreki M.F., Coffey A. (2005). Potential of the polyvalent anti-staphylococcus bacteriophage k for control of antibiotic-resistant *Staphylococci* from hospitals. Appl. Environ. Microbiol..

[31] Dakheel K.H., Rahim R.A., Neela V.K., Al-Obaidi J.R., Hun T.G., Isa M.N.M., Yusoff K. (2019). Genomic analyses of two novel biofilm-degrading methicillin-resistant *Staphylococcus aureus* phages. BMC Microbiol..

[32] Kaur S., Harjai K., Chhibber S. (2014). Bacteriophage mediated killing of *Staphylococcus aureus* in Vitro on orthopaedic K wires in presence of linezolid prevents implant colonization. PLoS ONE.

[33] Tkhilaishvili T., Lombardi L., Klatt A.-B., Trampuz A., Di Luca M. (2018). Bacteriophage Sb-1 enhances antibiotic activity against biofilm, degrades exopolysaccharide matrix and targets persisters of *Staphylococcus aureus*. Int. J. Antimicrob. Agents.

[34] Tkhilaishvili T., Wang L., Tavanti A., Trampuz A., Di Luca M. (2020). Antibacterial efficacy of two commercially available bacteriophage formulations, staphylococcal bacteriophage and PYO bacteriophage, against methicillin-resistant *Staphylococcus aureus*: Prevention and eradication of biofilm formation and control of a systemic infection of galleria mellonella larvae. Front. Microbiol..

[35] Jensen K.C., Hair B.B., Wienclaw T.M., Murdock M.H., Hatch J.B., Trent A.T., White T.D., Haskell K.J., Berges B.K. (2015). Isolation and host range of bacteriophage with lytic activity against methicillin-resistant *Staphylococcus aureus* and potential use as a fomite decontaminant. PLoS ONE.

[36] Zhou K., Li C., Chen D., Pan Y., Tao Y., Qu W., Liu Z., Wang X., Xie S. (2018). A review on nanosystems as an effective approach against infections of *Staphylococcus aureus*. Int. J. Nanomed..

[37] Labruère R., Sona A.J., Turos E. (2019). Anti-methicillin-resistant *Staphylococcus aureus* nanoantibiotics. Front. Pharmacol..

[38] Muppidi K., Pumerantz A.S., Wang J., Betageri G. (2012). Development and stability studies of novel liposomal vancomycin formulations. ISRN Pharm..

[39] Pumerantz A., Muppidi K., Agnihotri S., Guerra C., Venketaraman V., Wang J., Betageri G. (2011). Preparation of liposomal vancomycin and intracellular killing of meticillin-resistant *Staphylococcus aureus* (MRSA). Int. J. Antimicrob. Agents.

[40] Pumerantz A.S. (2012). PEGylated liposomal vancomycin: A glimmer of hope for improving treatment outcomes in MRSA pneumonia. Recent Pat. Anti-Infect Drug Disc..

[41] Sande L., Sanchez M., Montes J., Wolf A.J., Morgan M.A., Omri A., Liu G.Y. (2012). Liposomal encapsulation of vancomycin improves killing of methicillin-resistant *Staphylococcus aureus* in a murine infection model. J. Antimicrob. Chemother..

[42] Muppidi K., Wang J., Betageri G., Pumerantz A.S. (2011). PEGylated liposome encapsulation increases the lung tissue concentration of vancomycin. Antimicrob. Agents Chemother..

[43] Hajiahmadi F., Alikhani M.Y., Shariatifar H., Arabestani M.R., Ahmadvand D. (2019). The bactericidal effect of lysostaphin coupled with liposomal vancomycin as a dual combating system applied directly on methicillin-resistant *Staphylococcus aureus* infected skin wounds in mice. Int. J. Nanomed..

[44] Pornpattananangkul D., Zhang L., Olson S., Aryal S., Obonyo M., Vecchio K., Huang C.-M., Zhang L. (2011). Bacterial toxin-triggered drug release from gold nanoparticle-stabilized liposomes for the treatment of bacterial infection. J. Am. Chem. Soc..

[45] Scriboni A.B., Couto V.M., de Morais Ribeiro L.N., Freires I.A., Groppo F.C., de Paula E., Franz-Montan M., Cogo-Müller K. (2019). Fusogenic liposomes increase the antimicrobial activity of vancomycin against *Staphylococcus aureus* biofilm. Front. Pharmacol..

[46] Makhathini S.S., Kalhapure R.S., Jadhav M., Waddad A.Y., Gannimani R., Omolo C.A., Rambharose S., Mocktar C., Govender T. (2019). Novel two-chain fatty acid-based lipids for development of vancomycin PH-responsive liposomes against *Staphylococcus aureus* and methicillin-resistant *Staphylococcus aureus* (MRSA). J. Drug Target..

[47] Seedat N., Kalhapure R.S., Mocktar C., Vepuri S., Jadhav M., Soliman M., Govender T. (2016). Co-encapsulation of multi-lipids and polymers enhances the performance of vancomycin in lipid-polymer hybrid nanoparticles: In Vitro and in silico studies. Mater. Sci. Eng. C Mater. Biol. Appl..

[48] Garcia C.B., Shi D., Webster T.J. (2017). Tat-functionalized liposomes for the treatment of meningitis: An In Vitro study. Int. J. Nanomed..

[49] Jadhav M., Kalhapure R.S., Rambharose S., Mocktar C., Singh S., Kodama T., Govender T. (2018). Novel lipids with three C18-fatty acid chains and an amino acid head group for ph-responsive and sustained antibiotic delivery. Chem. Phys. Lipids.

[50] Omolo C.A., Megrab N.A., Kalhapure R.S., Agrawal N., Jadhav M., Mocktar C., Rambharose S., Maduray K., Nkambule B., Govender T. (2019). Liposomes with PH responsive “on and off” switches for targeted and intracellular delivery of antibiotics. J. Liposome Res..

[51] Thapa R.K., Kiick K.L., Sullivan M.O. (2020). Encapsulation of collagen mimetic peptide-tethered vancomycin liposomes in collagen-based scaffolds for infection control in wounds. Acta Biomater..

[52] Rukavina Z., Klarić M.Š., Filipović-Grčić J., Lovrić J., Vanić Ž. (2018). Azithromycin-loaded liposomes for enhanced topical treatment of methicillin-resistant *Staphyloccocus aureus* (MRSA) infections. Int. J. Pharm..

[53] Liu X., Li Z., Wang X., Chen Y., Wu F., Men K., Xu T., Luo Y., Yang L. (2016). Novel antimicrobial peptide-modified azithromycin-loaded liposomes against methicillin-resistant *Staphylococcus aureus*. Int. J. Nanomed..

[54] Hsu C.-Y., Yang S.-C., Sung C.T., Weng Y.-H., Fang J.-Y. (2017). Anti-MRSA malleable liposomes carrying chloramphenicol for ameliorating hair follicle targeting. Int. J. Nanomed..

[55] Ingebrigtsen S.G., Didriksen A., Johannessen M., Škalko-Basnet N., Holsæter A.M. (2017). Old drug, new wrapping—A possible comeback for chloramphenicol?. Int. J. Pharm..

[56] Jiang H., Xiong M., Bi Q., Wang Y., Li C. (2016). Self-enhanced targeted delivery of a cell wall- and membrane-active antibiotics, daptomycin, against staphylococcal pneumonia. Acta Pharm. Sin. B.

[57] Meng Y., Hou X., Lei J., Chen M., Cong S., Zhang Y., Ding W., Li G., Li X. (2016). Multi-functional liposomes enhancing target and antibacterial immunity for antimicrobial and anti-biofilm against methicillin-resistant *Staphylococcus aureus*. Pharm. Res..

[58] Alshamsan A., Aleanizy F.S., Badran M., Alqahtani F.Y., Alfassam H., Almalik A., Alosaimy S. (2019). Exploring anti-MRSA activity of chitosan-coated liposomal dicloxacillin. J. Microbiol. Methods.

[59] Shibata H., Nakano T., Parvez M.A.K., Furukawa Y., Tomoishi A., Niimi S., Arakaki N., Higuti T. (2009). Triple combinations of lower and longer alkyl gallates and oxacillin improve antibiotic synergy against methicillin-resistant *Staphylococcus aureus*. antimicrob. Agents Chemother..

[60] Lin M.-H., Hung C.-F., Aljuffali I.A., Sung C.T., Huang C.-T., Fang J.-Y. (2017). Cationic amphiphile in phospholipid bilayer or oil-water interface of nanocarriers affects planktonic and biofilm bacteria killing. Nanomed. Nanotechnol. Biol. Med..

[61] Koopmans T., Wood T.M., Hart P., Kleijn L.H.J., Hendrickx A.P.A., Willems R.J.L., Breukink E., Martin N.I. (2015). Semisynthetic lipopeptides derived from nisin display antibacterial activity and lipid ii binding on par with that of the parent compound. J. Am. Chem. Soc..

[62] Giordani B., Costantini P.E., Fedi S., Cappelletti M., Abruzzo A., Parolin C., Foschi C., Frisco G., Calonghi N., Cerchiara T. (2019). Liposomes containing biosurfactants isolated from lactobacillus gasseri exert antibiofilm activity against methicillin resistant *Staphylococcus aureus* strains. Eur. J. Pharm. Biopharm..

[63] Cui H., Li W., Li C., Vittayapadung S., Lin L. (2016). Liposome containing cinnamon oil with antibacterial activity against methicillin-resistant *Staphylococcus aureus* Biofilm. Biofouling.

[64] Cavalcanti I.M.F., Pontes-Neto J.G., Kocerginsky P.O., Bezerra-Neto A.M., Lima J.L.C., Lira-Nogueira M.C.B., Maciel M.A.V., Neves R.P., Pimentel M.F., Santos-Magalhães N.S. (2015). Antimicrobial activity of β-lapachone encapsulated into liposomes against meticillin-resistant *Staphylococcus aureus* and *Cryptococcus neoformans* clinical strains. J. Glob. Antimicrob. Resist..

[65] Gharib A., Faezizadeh Z., Godarzee M. (2013). Therapeutic efficacy of epigallocatechin gallate-loaded nanoliposomes against burn wound infection by methicillin-resistant *Staphylococcus aureus*. Skin Pharmacol. Physiol..

[66] Faezizadeh Z., Gharib A., Godarzee M. (2015). In-Vitro and In-Vivo evaluation of silymarin nanoliposomes against isolated methicillin-resistant *Staphylococcus aureus*. Iran. J. Pharm. Res..

[67] Huang C.-M., Chen C.-H., Pornpattananangkul D., Zhang L., Chan M., Hsieh M.-F., Zhang L. (2011). Eradication of drug resistant *Staphylococcus aureus* by liposomal oleic acids. Biomaterials.

[68] Wolfmeier H., Mansour S.C., Liu L.T., Pletzer D., Draeger A., Babiychuk E.B., Hancock R.E.W. (2018). Liposomal therapy attenuates dermonecrosis induced by community-associated methicillin-resistant *Staphylococcus aureus* by targeting α-type phenol-soluble modulins and α-hemolysin. EBioMedicine.

[69] Henry B., Neill D., Becker K.A., Gore S., Bricio-Moreno L., Ziobro R., Babiychuk E.B. (2015). Engineered liposomes sequester bacterial exotoxins and protect from severe invasive infections in mice. Nat. Biotechnol..

[70] Ferro S., Ricchelli F., Mancini G., Tognon G., Jori G. (2006). Inactivation of methicillin-resistant *Staphylococcus aureus* (MRSA) by liposome-delivered photosensitising agents. J. Photochem. Photobiol. B.

[71] Tsai T., Yang Y.-T., Wang T.-H., Chien H.-F., Chen C.-T. (2009). Improved photodynamic inactivation of gram-positive bacteria using hematoporphyrin encapsulated in liposomes and micelles. Lasers Surg. Med..

[72] Bombelli C., Bordi F., Ferro S., Giansanti L., Jori G., Mancini G., Mazzuca C., Monti D., Ricchelli F., Sennato S. (2008). New cationic liposomes as vehicles of M-tetrahydroxyphenylchlorin in photodynamic therapy of infectious diseases. Mol. Pharm..

[73] Yang K., Gitter B., Rüger R., Wieland G.D., Chen M., Liu X., Albrecht V., Fahr A. (2011). Antimicrobial peptide-modified liposomes for bacteria targeted delivery of temoporfin in photodynamic antimicrobial chemotherapy. Photochem. Photobiol. Sci..

[74] Ferro S., Ricchelli F., Monti D., Mancini G., Jori G. (2007). Efficient photoinactivation of methicillin-resistant *Staphylococcus aureus* by a novel porphyrin incorporated into a poly-cationic liposome. Int. J. Biochem. Cell Biol..

[75] Atashbeyk D.G., Khameneh B., Tafaghodi M., Bazzaz B.S.F. (2014). Eradication of methicillin-resistant *Staphylococcus aureus* infection by nanoliposomes loaded with gentamicin and oleic acid. Pharm. Biol..

[76] Bhise K., Sau S., Kebriaei R., Rice S.A., Stamper K.C., Alsaab H.O., Rybak M.J., Iyer A.K. (2018). Combination of vancomycin and cefazolin lipid nanoparticles for overcoming antibiotic resistance of MRSA. Mater. Basel Switz..

[77] Li Y., Su T., Zhang Y., Huang X., Li J., Li C. (2015). Liposomal co-delivery of daptomycin and clarithromycin at an optimized ratio for treatment of methicillin-resistant *Staphylococcus aureus* infection. Drug Deliv..

[78] Chhibber S., Kaur J., Kaur S. (2018). Liposome entrapment of bacteriophages improves wound healing in a diabetic mouse MRSA infection. Front. Microbiol..

[79] Meng J., Wang H., Hou Z., Chen T., Fu J., Ma X., He G., Xue X., Jia M., Luo X. (2009). Novel anion liposome-encapsulated antisense oligonucleotide restores susceptibility of methicillin-resistant *Staphylococcus aureus* and rescues mice from lethal sepsis by targeting MecA. Antimicrob. Agents Chemother..

[80] Hibbitts A., Lucía A., Serrano-Sevilla I., De Matteis L., McArthur M., de la Fuente J.M., Aínsa J.A., Navarro F. (2019). Co-delivery of free vancomycin and transcription factor decoy-nanostructured lipid carriers can enhance inhibition of methicillin resistant *Staphylococcus aureus* (MRSA). PLoS ONE.

[81] Alalaiwe A., Wang P.-W., Lu P.-L., Chen Y.-P., Fang J.-Y., Yang S.-C. (2018). Synergistic anti-MRSA activity of cationic nanostructured lipid carriers in combination with oxacillin for cutaneous application. Front. Microbiol..

[82] Umerska A., Cassisa V., Bastiat G., Matougui N., Nehme H., Manero F., Eveillard M., Saulnier P. (2017). Synergistic interactions between antimicrobial peptides derived from plectasin and lipid nanocapsules containing monolaurin as a cosurfactant against *Staphylococcus aureus*. Int. J. Nanomed..

[83] Sonawane S.J., Kalhapure R.S., Rambharose S., Mocktar C., Vepuri S.B., Soliman M., Govender T. (2016). Ultra-small lipid-dendrimer hybrid nanoparticles as a promising strategy for antibiotic delivery: In Vitro and in silico studies. Int. J. Pharm..

[84] Akram F.E., El-Tayeb T., Abou-Aisha K., El-Azizi M. (2016). A combination of silver nanoparticles and visible blue light enhances the antibacterial efficacy of ineffective antibiotics against methicillin-resistant *Staphylococcus aureus* (MRSA). Ann. Clin. Microbiol. Antimicrob..

[85] Masri A., Anwar A., Ahmed D., Siddiqui R.B., Shah M.R., Khan N.A. (2018). Silver nanoparticle conjugation-enhanced antibacterial efficacy of clinically approved drugs cephradine and vildagliptin. Antibiotics.

[86] Farooq U., Ahmad T., Khan A., Sarwar R., Shafiq J., Raza Y., Ahmed A., Ullah S., Ur Rehman N., Al-Harrasi A. (2019). Rifampicin conjugated silver nanoparticles: A new arena for development of antibiofilm potential against methicillin resistant *Staphylococcus aureus* and klebsiella pneumoniae. Int. J. Nanomed..

[87] Figueiredo E.P., Ribeiro J.M., Nishio E.K., Scandorieiro S., Costa A.F., Cardozo V.F., Oliveira A.G., Durán N., Panagio L.A., Kobayashi R. (2019). New approach for simvastatin as an antibacterial: Synergistic effect with bio-synthesized silver nanoparticles against multidrug-resistant bacteria. Int. J. Nanomed..

[88] Halawani E.M., Hassan A.M., Gad El-Rab S.M.F. (2020). Nanoformulation of biogenic cefotaxime-conjugated-silver nanoparticles for enhanced antibacterial efficacy against multidrug-resistant bacteria and anticancer studies. Int. J. Nanomed..

[89] Xu N., Cheng H., Xu J., Li F., Gao B., Li Z., Gao C., Huo K., Fu J., Xiong W. (2017). Silver-loaded nanotubular structures enhanced bactericidal efficiency of antibiotics with synergistic effect In Vitro and In Vivo. Int. J. Nanomed..

[90] Zhang P., Qin J., Zhang B., Zheng Y., Yang L., Shen Y., Zuo B., Zhang F. (2019). Gentamicin-loaded silk/nanosilver composite scaffolds for MRSA-induced chronic osteomyelitis. R. Soc. Open Sci..

[91] Perni S., Prokopovich P. (2014). Continuous release of gentamicin from gold nanocarriers. RSC Adv..

[92] Meeker D.G., Wang T., Harrington W.N., Zharov V.P., Johnson S.A., Jenkins S.V., Oyibo S.E., Walker C.M., Mills W.B., Shirtliff M.E. (2018). Versatility of targeted antibiotic-loaded gold nanoconstructs for the treatment of biofilm-associated bacterial infections. Int. J. Hyperth..

[93] de Carvalho J.F., de Azevedo Í.M., Rocha K.B.F., Medeiros A.C., Carriço A.D.S. (2017). Oxacillin magnetically targeted for the treatment of methicillin-resistant *S. aureus* infection in rats. Acta Circ. Bras..

[94] Mosselhy D.A., He W., Hynönen U., Meng Y., Mohammadi P., Palva A., Feng Q., Hannula S.-P., Nordström K., Linder M.B. (2018). Silica-gentamicin nanohybrids: Combating antibiotic resistance, bacterial biofilms, and in Vivo toxicity. Int. J. Nanomed..

[95] Cihalova K., Chudobova D., Michalek P., Moulick A., Guran R., Kopel P., Adam V., Kizek R. (2015). *Staphylococcus aureus* and MRSA growth and biofilm formation after treatment with antibiotics and SeNPs. Int. J. Mol. Sci..

[96] Saidykhan L., Bakar M.Z.B.A., Rukayadi Y., Kura A.U., Latifah S.Y. (2016). Development of nanoantibiotic delivery system using cockle shell-derived aragonite nanoparticles for treatment of osteomyelitis. Int. J. Nanomed..

[97] Hussein-Al-Ali S.H., Zowalaty M.E.E., Hussein M.Z., Ismail M., Webster T.J. (2014). Synthesis, characterization, controlled release, and antibacterial studies of a novel streptomycin chitosan magnetic nanoantibiotic. Int. J. Nanomed..

[98] Mushtaq S., Khan J.A., Rabbani F., Latif U., Arfan M., Yameen M.A. (2017). Biocompatible biodegradable polymeric antibacterial nanoparticles for enhancing the effects of a third-generation cephalosporin against resistant bacteria. J. Med. Microbiol..

[99] Duncan B., Li X., Landis R.F., Kim S.T., Gupta A., Wang L.-S., Ramanathan R., Tang R., Boerth J.A., Rotello V.M. (2015). Nanoparticle stabilized capsules for the treatment of bacterial biofilms. ACS Nano.

[100] Turos E., Shim J.-Y., Wang Y., Greenhalgh K., Reddy G.S.K., Dickey S., Lim D.V. (2007). Antibiotic-conjugated polyacrylate nanoparticles: New opportunities for development of anti-mrsa agents. Bioorg. Med. Chem. Lett..

[101] Turos E., Reddy G.S.K., Greenhalgh K., Ramaraju P., Abeylath S.C., Jang S., Dickey S., Lim D.V. (2007). Penicillin-bound polyacrylate nanoparticles: Restoring the activity of beta-lactam antibiotics against MRSA. Bioorg. Med. Chem. Lett..

[102] Kalita S., Devi B., Kandimalla R., Sharma K.K., Sharma A., Kalita K., Kataki A.C., Kotoky J. (2015). Chloramphenicol encapsulated in poly-ε-caprolactone-pluronic composite: Nanoparticles for treatment of mrsa-infected burn wounds. Int. J. Nanomed..

[103] Qiu Y., Wu Y., Lu B., Zhu G., Gong T., Wang R., Peng Q., Li Y. (2020). Inhibition of methicillin-resistant *Staphylococcus aureus* (MRSA) biofilm by cationic poly (D, L-Lactide-Co-Glycolide) nanoparticles. Biofouling.

[104] Hasan N., Cao J., Lee J., Hlaing S.P., Oshi M.A., Naeem M., Ki M.-H., Lee B.L., Jung Y., Yoo J.-W. (2019). Bacteria-targeted clindamycin loaded polymeric nanoparticles: Effect of surface charge on nanoparticle adhesion to MRSA, antibacterial activity, and wound healing. Pharmaceutics.

[105] Mir M., Ahmed N., Permana A.D., Rodgers A.M., Donnelly R.F., Rehman A.U. (2019). Enhancement in site-specific delivery of carvacrol against methicillin resistant *Staphylococcus aureus* induced skin infections using enzyme responsive nanoparticles: A Proof of concept study. Pharmaceutics.

[106] Yousry C., Elkheshen S.A., El-Laithy H.M., Essam T., Fahmy R.H. (2017). Studying the influence of formulation and process variables on vancomycin-loaded polymeric nanoparticles as potential carrier for enhanced ophthalmic delivery. Eur. J. Pharm. Sci..

[107] Pei Y., Mohamed M.F., Seleem M.N., Yeo Y. (2017). Particle engineering for intracellular delivery of vancomycin to methicillin-resistant *Staphylococcus aureus* (MRSA)-infected macrophages. J. Control. Release.

[108] Fiore E., Van Tyne D., Gilmore M.S. (2019). Pathogenicity of *Enterococci*. Microbiol. Spectr..

[109] Papadimitriou-Olivgeris M., Kolonitsiou F., Karamouzos V., Tsilipounidaki K., Nikolopoulou A., Fligou F., Marangos M., Petinaki E., Spiliopoulou I. (2020). Molecular characteristics and predictors of mortality among gram-positive bacteria isolated from bloodstream infections in critically ill patients during a 5-year period (2012–2016). Eur. J. Clin. Microbiol. Infect. Dis..

[110] Raza T., Ullah S.R., Mehmood K., Andleeb S. (2018). Vancomycin resistant *Enterococci*: A brief review. J. Pak. Med. Assoc..

[111] Al-Zubidi M., Widziolek M., Court E.K., Gains A.F., Smith R.E., Ansbro K., Alrafaie A., Evans C., Murdoch C., Mesnage S. (2019). Identification of novel bacteriophages with therapeutic potential that target *Enterococcus faecalis*. Infect. Immun..

[112] Khalifa L., Shlezinger M., Beyth S., Houri-Haddad Y., Coppenhagen-Glazer S., Beyth N., Hazan R. (2016). Phage therapy against *Enterococcus faecalis* in dental root canals. J. Oral Microbiol..

[113] Iram S., Akbar Khan J., Aman N., Nadhman A., Zulfiqar Z., Yameen M.A. (2016). Enhancing the anti-enterococci activity of different antibiotics by combining with metal oxide nanoparticles. Jundishapur J. Microbiol..

[114] Gu H., Ho P.-L., Tsang K.W.T., Wang L., Xu B. (2003). Using biofunctional magnetic nanoparticles to capture vancomycin-resistant *Enterococci* and other gram-positive bacteria at ultralow concentration. J. Am. Chem. Soc..

[115] Esmaeillou M., Zarrini G., Rezaee M.A., Mojarrad J.S., Bahadori A. (2017). Vancomycin capped with silver nanoparticles as an antibacterial agent against multi-drug resistance bacteria. Adv. Pharm. Bull..

[116] Lam A.H.C., Sandoval N., Wadhwa R., Gilkes J., Do T.Q., Ernst W., Chiang S.-M., Kosina S., Howard Xu H., Fujii G. (2016). Assessment of free fatty acids and cholesteryl esters delivered in liposomes as novel class of antibiotic. BMC Res. Notes.

[117] Li J., Zhang K., Ruan L., Chin S.F., Wickramasinghe N., Liu H., Ravikumar V., Ren J., Duan H., Yang L. (2018). Block copolymer nanoparticles remove biofilms of drug-resistant gram-positive bacteria by nanoscale bacterial debridement. Nano Lett..

[118] Czepiel J., Dróżdż M., Pituch H., Kuijper E.J., Perucki W., Mielimonka A., Goldman S., Wultańska D., Garlicki A., Biesiada G. (2019). *Clostridium difficile* infection: Review. Eur. J. Clin. Microbiol. Infect. Dis..

[119] Rottner K., Stradal T.E.B., Wehland J. (2005). Bacteria-host-cell interactions at the plasma membrane: Stories on actin cytoskeleton subversion. Dev. Cell.

[120] Louie T.J., Meddings J. (2004). *Clostridium difficile* infection in hospitals: Risk factors and responses. Can. Med. Assoc. J. J. Assoc. Medicale Can..

[121] McDonald L.C., Gerding D.N., Johnson S., Bakken J.S., Carroll K.C., Coffin S.E., Dubberke E.R., Garey K.W., Gould C.V., Kelly C. (2018). Clinical practice guidelines for *Clostridium difficile* infection in adults and children: 2017 Update by the Infectious Diseases Society of America (IDSA) and Society for Healthcare Epidemiology of America (SHEA). Clin. Infect. Dis..

[122] Fortier L.-C. (2018). Bacteriophages contribute to shaping *Clostridioides (Clostridium) difficile* species. Front. Microbiol..

[123] Payne R.J., Phil D., Jansen V.A. (2000). Phage therapy: The peculiar kinetics of self-replicating pharmaceuticals. Clin. Pharmacol. Ther..

[124] Fu W., Forster T., Mayer O., Curtin J.J., Lehman S.M., Donlan R.M. (2010). Bacteriophage cocktail for the prevention of biofilm formation by *Pseudomonas aeruginosa* on catheters in an In Vitro model system. Antimicrob. Agents Chemother..

[125] Nale J.Y., Spencer J., Hargreaves K.R., Buckley A.M., Trzepiński P., Douce G.R., Clokie M.R.J. (2016). Bacteriophage combinations significantly reduce *Clostridium difficile* growth In Vitro and proliferation In Vivo. Antimicrob. Agents Chemother..

[126] Moelling K., Broecker F. (2016). Fecal microbiota transplantation to fight *Clostridium difficile* infections and other intestinal diseases. Bacteriophage.

[127] Zuo T., Wong S.H., Lam K., Lui R., Cheung K., Tang W., Ching J.Y.L., Chan P.K.S., Chan M.C.W., Wu J.C.Y. (2018). Bacteriophage transfer during faecal microbiota transplantation in *Clostridium difficile* infection is associated with treatment outcome. Gut.

[128] Chen Y.-H., Li T.-J., Tsai B.-Y., Chen L.-K., Lai Y.-H., Li M.-J., Tsai C.-Y., Tsai P.-J., Shieh D.-B. (2019). Vancomycin-loaded nanoparticles enhance sporicidal and antibacterial efficacy for *Clostridium difficile* infection. Front. Microbiol..

[129] Preisig D., Roth R., Tognola S., Varum F.J.O., Bravo R., Cetinkaya Y., Huwyler J., Puchkov M. (2016). Mucoadhesive microparticles for local treatment of gastrointestinal diseases. Eur. J. Pharm. Biopharm..

[130] Hegarty J.P., Krzeminski J., Sharma A.K., Guzman-Villanueva D., Weissig V., Stewart D.B. (2016). Bolaamphiphile-based nanocomplex delivery of phosphorothioate gapmer antisense oligonucleotides as a treatment for *Clostridium difficile*. Int. J. Nanomed..

[131] Bland S.D., Venable E.B., McPherson J.L., Atkinson R.L. (2017). Effects of liposomal-curcumin on five opportunistic bacterial strains found in the equine hindgut—Preliminary study. J. Anim. Sci. Technol..

[132] Aslam B., Rasool M., Muzammil S., Siddique A.B., Nawaz Z., Shafique M., Zahoor M.A., Binyamin R., Waseem M., Khurshid M. (2020). Carbapenem resistance: Mechanisms and drivers of global menace. Pathog. Bact..

[133] Papadimitriou-Olivgeris M., Fligou F., Bartzavali C., Zotou A., Spyropoulou A., Koutsileou K., Vamvakopoulou S., Sioulas N., Karamouzos V., Anastassiou E.D. (2017). Carbapenemase-producing *Klebsiella pneumoniae* bloodstream infection in critically ill patients: Risk factors and predictors of mortality. Eur. J. Clin. Microbiol. Infect. Dis..

[134] Nordmann P., Naas T., Poirel L. (2011). Global spread of carbapenemase-producing *Enterobacteriaceae*. Emerg. Infect. Dis..

[135] Yigit H., Queenan A.M., Anderson G.J., Domenech-Sanchez A., Biddle J.W., Steward C.D., Alberti S., Bush K., Tenover F.C. (2001). Novel carbapenem-hydrolyzing beta-lactamase, KPC-1, from a carbapenem-resistant strain of *Klebsiella pneumoniae*. Antimicrob. Agents Chemother..

[136] Elshamy A.A., Aboshanab K.M. (2020). A review on bacterial resistance to carbapenems: Epidemiology, detection and treatment options. Future Sci. OA.

[137] European Centre for Disease Prevention and Control Rapid Risk Assessment: Plasmid-Mediated Colistin Resistance in Enterobacteriaceae, 15 June 2016. https://www.ecdc.europa.eu/en/publications-data/rapid-risk-assessment-plasmid-mediated-colistin-resistance-enterobacteriaceae-15.

[138] Munro N. (2015). Antimicrobial resistance: Thinking outside the box. AACN Adv. Crit. Care.

[139] Al-Jassim N., Mantilla-Calderon D., Scarascia G., Hong P.-Y. (2018). Bacteriophages to sensitize a pathogenic new delhi metallo β-lactamase-positive *Escherichia coli* to solar disinfection. Environ. Sci. Technol..

[140] Oliveira H., Pinto G., Oliveira A., Oliveira C., Faustino M.A., Briers Y., Domingues L., Azeredo J. (2016). Characterization and genome sequencing of a citrobacter freundii phage cfp1 harboring a lysin active against multidrug-resistant isolates. Appl. Microbiol. Biotechnol..

[141] Santiago A.J., Burgos-Garay M.L., Kartforosh L., Mazher M., Donlan R.M. (2020). Bacteriophage treatment of carbapenemase-producing *Klebsiella pneumoniae* in a multispecies biofilm: A potential biocontrol strategy for healthcare facilities. AIMS Microbiol..

[142] Ciacci N., D’Andrea M.M., Marmo P., Demattè E., Amisano F., Di Pilato V., Fraziano M., Lupetti P., Rossolini G.M., Thaller M.C. (2018). Characterization of VB_Kpn_F48, a newly discovered lytic bacteriophage for *Klebsiella pneumoniae* of sequence type 101. Viruses.

[143] González-Paredes A., Sitia L., Ruyra A., Morris C.J., Wheeler G.N., McArthur M., Gasco P. (2019). Solid lipid nanoparticles for the delivery of anti-microbial oligonucleotides. Eur. J. Pharm. Biopharm..

[144] Mhango E.K.G., Kalhapure R.S., Jadhav M., Sonawane S.J., Mocktar C., Vepuri S., Soliman M., Govender T. (2017). Preparation and optimization of meropenem-loaded solid lipid nanoparticles: In Vitro evaluation and molecular modeling. AAPS PharmSciTech.

[145] Panáček A., Smékalová M., Večeřová R., Bogdanová K., Röderová M., Kolář M., Kilianová M., Hradilová Š., Froning J.P., Havrdová M. (2016). Silver nanoparticles strongly enhance and restore bactericidal activity of inactive antibiotics against multiresistant *Enterobacteriaceae*. Colloids Surf. B Biointerfaces.

[146] Scandorieiro S., de Camargo L.C., Lancheros C.A.C., Yamada-Ogatta S.F., Nakamura C.V., de Oliveira A.G., Andrade C.G.T.J., Duran N., Nakazato G., Kobayashi R.K.T. (2016). Synergistic and additive effect of oregano essential oil and biological silver nanoparticles against multidrug-resistant bacterial strains. Front. Microbiol..

[147] Kumar N., Kumar G., Mallick S., Ghosh S.K., Pramanick P., Ghosh A.S. (2019). Bio-surfactin stabilised silver nanoparticles exert inhibitory effect over New-Delhi Metallo-Beta-Lactamases (NDMs) and the cells harbouring NDMs. FEMS Microbiol. Lett..

[148] Bharathan S., Sundaramoorthy N.S., Chandrasekaran H., Rangappa G., ArunKumar G., Subramaniyan S.B., Veerappan A., Nagarajan S. (2019). Sub lethal levels of platinum nanoparticle cures plasmid and in combination with carbapenem, curtails carbapenem resistant *Escherichia coli*. Sci. Rep..

[149] Shaker M.A., Shaaban M.I. (2017). Formulation of carbapenems loaded gold nanoparticles to combat multi-antibiotic bacterial resistance: In Vitro antibacterial study. Int. J. Pharm..

[150] Abdelkader A., El-Mokhtar M.A., Abdelkader O., Hamad M.A., Elsabahy M., El-Gazayerly O.N. (2017). Ultrahigh antibacterial efficacy of meropenem-loaded chitosan nanoparticles in a septic animal model. Carbohydr. Polym..

[151] Fazli Y., Shariatinia Z., Kohsari I., Azadmehr A., Pourmortazavi S.M. (2016). A novel chitosan-polyethylene oxide nanofibrous mat designed for controlled co-release of hydrocortisone and imipenem/cilastatin drugs. Int. J. Pharm..

[152] Nandakumar V., Geetha V., Chittaranjan S., Doble M. (2013). High glycolic poly (DL Lactic Co Glycolic Acid) nanoparticles for controlled release of meropenem. Biomed. Pharmacother. Biomed. Pharmacother..

[153] Horcajada J.P., Montero M., Oliver A., Sorlí L., Luque S., Gómez-Zorrilla S., Benito N., Grau S. (2019). Epidemiology and treatment of multidrug-resistant and extensively drug-resistant *Pseudomonas aeruginosa* infections. Clin. Microbiol. Rev..

[154] Oliver A., Mulet X., López-Causapé C., Juan C. (2015). The increasing threat of *Pseudomonas aeruginosa* high-risk clones. Drug Resist. Updat. Rev. Comment. Antimicrob. Anticancer Chemother..

[155] Bassetti M., Vena A., Croxatto A., Righi E., Guery B. (2018). How to manage *Pseudomonas aeruginosa* infections. Drugs Context.

[156] Wang J., Hu B., Xu M., Yan Q., Liu S., Zhu X., Sun Z., Reed E., Ding L., Gong J. (2006). Use of bacteriophage in the treatment of experimental animal bacteremia from imipenem-resistant pseudomonas aeruginosa. Int. J. Mol. Med..

[157] Shivshetty N., Hosamani R., Ahmed L., Oli A.K., Sannauallah S., Sharanbassappa S., Patil S.A., Kelmani C.R. (2014). Experimental protection of diabetic mice against lethal, P. aeruginosa infection by bacteriophage. BioMed Res. Int..

[158] Larché J., Pouillot F., Essoh C., Libisch B., Straut M., Lee J.C., Soler C., Lamarca R., Gleize E., Gabard J. (2012). Rapid identification of international multidrug-resistant *Pseudomonas aeruginosa* clones by multiple-locus variable number of tandem repeats analysis and investigation of their susceptibility to lytic bacteriophages. Antimicrob. Agents Chemother..

[159] Drulis-Kawa Z., Gubernator J., Dorotkiewicz-Jach A., Doroszkiewicz W., Kozubek A. (2006). In Vitro antimicrobial activity of liposomal meropenem against *Pseudomonas aeruginosa* strains. Int. J. Pharm..

[160] Zahra M.-J., Hamed H., Mohammad R.-Y., Nosratollah Z., Akbarzadeh A., Morteza M. (2017). Evaluation and study of antimicrobial activity of nanoliposomal meropenem against *Pseudomonas aeruginosa* isolates. Artif. Cells Nanomed. Biotechnol..

[161] Ahmady I.M., Hameed M.K., Almehdi A.M., Arooj M., Workie B., Sahle-Demessie E., Han C., Mohamed A.A. (2019). Green and cytocompatible carboxyl modified gold-lysozyme nanoantibacterial for combating multidrug-resistant superbugs. Biomater. Sci..

[162] Khataminejad M.R., Mirnejad R., Sharif M., Hashemi M., Sajadi N., Piranfar V. (2015). Antimicrobial effect of imipenem-functionalized Fe_2_O_3_ nanoparticles on *Pseudomonas aeruginosa* producing metallo β-lactamases. Iran. J. Biotechnol..

[163] Lob S.H., Hoban D.J., Sahm D.F., Badal R.E. (2016). Regional differences and trends in antimicrobial susceptibility of acinetobacter baumannii. Int. J. Antimicrob. Agents.

[164] Harding C.M., Hennon S.W., Feldman M.F. (2018). Uncovering the mechanisms of *Acinetobacter baumannii* virulence. Nat. Rev. Microbiol..

[165] Perez F., Hujer A.M., Hujer K.M., Decker B.K., Rather P.N., Bonomo R.A. (2007). Global challenge of multidrug-resistant *Acinetobacter baumannii*. Antimicrob. Agents Chemother..

[166] Jeon J., D’Souza R., Pinto N., Ryu C.-M., Park J., Yong D., Lee K. (2016). Characterization and complete genome sequence analysis of two myoviral bacteriophages infecting clinical carbapenem-resistant *Acinetobacter baumannii* isolates. J. Appl. Microbiol..

[167] Ho Y.-H., Tseng C.-C., Wang L.-S., Chen Y.-T., Ho G.-J., Lin T.-Y., Wang L.-Y., Chen L.-K. (2016). Application of bacteriophage-containing aerosol against nosocomial transmission of carbapenem-resistant *Acinetobacter baumannii* in an intensive care unit. PLoS ONE.

[168] Leshkasheli L., Kutateladze M., Balarjishvili N., Bolkvadze D., Save J., Oechslin F., Que Y.-A., Resch G. (2019). Efficacy of newly isolated and highly potent bacteriophages in a mouse model of extensively drug-resistant *Acinetobacter baumannii* bacteraemia. J. Glob. Antimicrob. Resist..

[169] Jansen M., Wahida A., Latz S., Krüttgen A., Häfner H., Buhl E.M., Ritter K., Horz H.-P. (2018). Enhanced antibacterial effect of the novel T4-like bacteriophage KARL-1 in combination with antibiotics against multi-drug resistant *Acinetobacter baumannii*. Sci. Rep..

[170] Zendegani E., Dolatabadi S. (2020). The efficacy of imipenem conjugated with synthesized silver nanoparticles against *Acinetobacter baumannii* clinical isolates, Iran. Biol. Trace Elem. Res..

[171] Wan G., Ruan L., Yin Y., Yang T., Ge M., Cheng X. (2016). Effects of silver nanoparticles in combination with antibiotics on the resistant bacteria *Acinetobacter baumannii*. Int. J. Nanomed..

[172] Javed A., Parvaiz F., Manzoor S. (2019). Bacterial vaginosis: An insight into the prevalence, alternative treatments regimen and it’s associated resistance patterns. Microb. Pathog..

[173] Kirkcaldy R.D., Weston E., Segurado A.C., Hughes G. (2019). Epidemiology of *Gonorrhoea*: A global perspective. Sex. Health.

[174] Rowley J., Vander Hoorn S., Korenromp E., Low N., Unemo M., Abu-Raddad L.J., Chico R.M., Smolak A., Newman L., Gottlieb S. (2019). Chlamydia, gonorrhoea, trichomoniasis and syphilis: Global prevalence and incidence estimates, 2016. Bull. World Health Organ..

[175] da Costa-Lourenço A.P.R., Santos K.T.B.D., Moreira B.M., Fracalanzza S.E.L., Bonelli R.R. (2017). Antimicrobial resistance in *Neisseria gonorrhoeae*: History, molecular mechanisms and epidemiological aspects of an emerging global threat. Braz. J. Microbiol..

[176] Belkacem A., Jacquier H., Goubard A., Mougari F., La Ruche G., Patey O., Micaëlo M., Semaille C., Cambau E., Bercot B. (2016). Molecular epidemiology and mechanisms of resistance of azithromycin-resistant *Neisseria gonorrhoeae* isolated in france during 2013–14. J. Antimicrob. Chemother..

[177] Cern A., Connolly K.L., Jerse A.E., Barenholz Y. (2018). In Vitro susceptibility of *Neisseria gonorrhoeae* strains to mupirocin, an antibiotic reformulated for parenteral administration in nanoliposomes. Antimicrob. Agents Chemother..

[178] Wang L., Sassi A.B., Patton D., Isaacs C., Moncla B.J., Gupta P., Rohan L.C. (2012). Development of a liposome microbicide formulation for vaginal delivery of octylglycerol for HIV prevention. Drug Dev. Ind. Pharm..

[179] Su W.-H., Ho T.-Y., Tsou T.-S., Lee W.-L., Wang K.-C., Yu Y.-Y., Chen T.-J., Tan C.-H., Kuo C.-D., Chen C.-S. (2013). Development of a chip-based multiplexed immunoassay using liposomal nanovesicles and its application in the detection of pathogens causing female lower genital tract infections. Taiwan. J. Obstet. Gynecol..

[180] Gala R.P., Zaman R.U., D’Souza M.J., Zughaier S.M. (2018). Novel whole-cell inactivated *Neisseria gonorrhoeae* microparticles as vaccine formulation in microneedle-based transdermal immunization. Vaccines.

[181] Li L.-H., Yen M.-Y., Ho C.-C., Wu P., Wang C.-C., Maurya P.K., Chen P.-S., Chen W., Hsieh W.-Y., Chen H.-W. (2013). Non-cytotoxic nanomaterials enhance antimicrobial activities of cefmetazole against multidrug-resistant *Neisseria gonorrhoeae*. PLoS ONE.

[182] WHO (2020). Global Tuberculosis Report. https://apps.who.int/iris/bitstream/handle/10665/336069/9789240013131-eng.pdf.

[183] Onyango R. (2011). State of the globe: Tracking tuberculosis is the test of time. J. Glob. Infect. Dis..

[184] Vyas S.P., Kannan M.E., Jain S., Mishra V., Singh P. (2004). Design of liposomal aerosols for improved delivery of rifampicin to alveolar macrophages. Int. J. Pharm..

[185] Pinheiro M., Lúcio M., Lima J.L., Reis S. (2011). Liposomes as drug delivery systems for the treatment of TB. Nanomedicine.

[186] Carrigy N.B., Larsen S.E., Reese V., Pecor T., Harrison M., Kuehl P.J., Hatfull G.F., Sauvageau D., Baldwin S.L., Finlay W.H. (2019). Prophylaxis of *Mycobacterium tuberculosis* H37Rv infection in a preclinical mouse model via inhalation of nebulized bacteriophage D29. Antimicrob. Agents Chemother..

[187] Carrigy N.B., Chang R.Y., Leung S.S.Y., Harrison M., Petrova Z., Pope W.H., Hatfull G.F., Britton W.J., Chan H.K., Sauvageau D. (2017). Anti-tuberculosis bacteriophage D29 delivery with a vibrating mesh nebulizer, jet nebulizer, and soft mist inhaler. Pharm. Res..

[188] Ly A., Carrigy N.B., Wang H., Harrison M., Sauvageau D., Martin A.R., Vehring R., Finlay W.H. (2019). Atmospheric spray freeze drying of sugar solution with phage D29. Front. Microbiol..

[189] Kelly C., Jefferies C., Cryan S.-A. (2011). Targeted liposomal drug delivery to monocytes and macrophages. J. Drug Deliv..

[190] van Zyl L., Viljoen J.M., Haynes R.K., Aucamp M., Ngwane A.H., du Plessis J. (2019). Topical delivery of artemisone, clofazimine and decoquinate encapsulated in vesicles and their in vitro efficacy against *Mycobacterium tuberculosis*. AAPS Pharm. Sci. Tech..

[191] Gaidukevich S.K., Mikulovich Y.L., Smirnova T.G., Andreevskaya S.N., Sorokoumova G.M., Chernousova L.N., Selishcheva A.A., Shvets V.I. (2016). Antibacterial effects of liposomes containing phospholipid cardiolipin and fluoroquinolone levofloxacin on *Mycobacterium tuberculosis* with extensive drug resistance. Bull. Exp. Biol. Med..

[192] Lira M.C.B., Siqueira-Moura M.P., Rolim-Santos H.M.L., Galetti F.C.S., Simioni A.R., Santos N.P., Tabosa Do Egito E.S., Silva C.L., Tedesco A.C., Santos-Magalhães N.S. (2009). In vitrouptake and antimycobacterial activity of liposomal usnic acid formulation. J. Liposome Res..

[193] Ferraz-Carvalho R.S., Pereira M.A., Linhares L.A., Lira-Nogueira M.C.B., Cavalcanti I.M.F., Santos-Magalhães N.S., Montenegro L.M.L. (2016). Effects of the encapsulation of usnic acid into liposomes and interactions with antituberculous agents against multidrug-resistant tuberculosis clinical isolates. Mem. Inst. Oswaldo Cruz.

[194] Miretti M., Juri L., Cosiansi M.C., Tempesti T.C., Baumgartner M.T. (2019). Antimicrobial effects of ZnPc delivered into liposomes on multidrug resistant (MDR)-*Mycobacterium tuberculosis*. ChemistrySelect.

[195] Mata-Espinosa D., Molina-Salinas G.M., Barrios-Payán J., Navarrete-Vázquez G., Marquina B., Ramos-Espinosa O., Bini E.I., Baeza I., Hernández-Pando R. (2015). Therapeutic efficacy of liposomes containing 4-(5-pentadecyl-1,3,4-oxadiazol-2-yl) pyridine in a murine model of progressive pulmonary tuberculosis. Pulm. Pharmacol. Ther..

[196] Labana S., Pandey R., Sharma S., Khuller G.K. (2002). Chemotherapeutic activity against murine tuberculosis of once weekly administered drugs (isoniazid and rifampicin) encapsulated in liposomes. Int. J. Antimicrob. Agents.

[197] Gaspar M.M., Cruz A., Penha A.F., Reymão J., Sousa A.C., Eleutério C.V., Domingues S.A., Fraga A.G., Filho A.L., Cruz M.E.M. (2008). Rifabutin encapsulated in liposomes exhibits increased therapeutic activity in a model of disseminated tuberculosis. Int. J. Antimicrob. Agents.

[198] Shkurupy V.A., Cherdantseva L.A., Kovner A.V., Troitskii A.V., Bystrova T.N., Starostenko A.A. (2020). Structural changes in the lungs and liver of mice with experimental tuberculosis treated with liposome-encapsulated dextrazide. Bull. Exp. Biol. Med..

[199] El-Ridy M.S., Mostafa D.M., Shehab A., Nasr E.A., Abd El-Alim S. (2007). Biological evaluation of pyrazinamide liposomes for treatment of *Mycobacterium tuberculosis*. Int. J. Pharm..

[200] Dhillon J., Fielding R., Adler-Moore J., Goodall R.L., Mitchison D. (2001). The activity of low-clearance liposomal amikacin in experimental murine tuberculosis. J. Antimicrob. Chemother..

[201] Pandey R., Khuller G.K. (2004). Subcutaneous nanoparticle-based antitubercular chemotherapy in an experimental model. J. Antimicrob. Chemother..

[202] Pandey R., Zahoor A., Sharma S., Khuller G.K. (2003). Nanoparticle encapsulated antitubercular drugs as a potential oral drug delivery system against murine tuberculosis. Tuberculosis.

[203] Sharma A., Sharma S., Khuller G.K. (2004). Lectin-functionalized poly (lactide-co-glycolide) nanoparticles as oral/aerosolized antitubercular drug carriers for treatment of tuberculosis. J. Antimicrob. Chemother..

[204] Pandey R., Sharma A., Zahoor A., Sharma S., Khuller G.K., Prasad B. (2003). Poly (DL-lactide-co-glycolide) nanoparticle-based inhalable sustained drug delivery system for experimental tuberculosis. J. Antimicrob. Chemother..

[205] Sung J.C., Padilla D.J., Garcia-Contreras L., Verberkmoes J.L., Durbin D., Peloquin C.A., Elbert K.J., Hickey A.J., Edwards D.A. (2009). Formulation and pharmacokinetics of self-assembled rifampicin nanoparticle systems for pulmonary delivery. Pharm. Res..

[206] Kumar G., Sharma S., Shafiq N., Pandhi P., Khuller G.K., Malhotra S. (2011). Pharmacokinetics and tissue distribution studies of orally administered nanoparticles encapsulated ethionamide used as potential drug delivery system in management of multi-drug resistant tuberculosis. Drug Deliv..

[207] Sosunov V., Mischenko V., Eruslanov B., Svetoch E., Shakina Y., Stern N., Majorov K., Sorokoumova G., Selishcheva A., Apt A. (2007). Antimycobacterial activity of bacteriocins and their complexes with liposomes. J. Antimicrob. Chemother..

[208] Moradi S., Taran M., Mohajeri P., Sadrjavadi K., Sarrami F., Karton A., Shahlaei M. (2018). Study of dual encapsulation possibility of hydrophobic and hydrophilic drugs into a nanocarrier based on bio-polymer coated graphene oxide using density functional theory, molecular dynamics simulation and experimental methods. J. Mol. Liq..

[209] Jahagirdar P.S., Gupta P.K., Kulkarni S.P., Devarajan P.V. (2020). Intramacrophage delivery of dual drug loaded nanoparticles for effective clearance of *Mycobacterium tuberculosis*. J. Pharm. Sci..

[210] Upadhyay T.K., Fatima N., Sharma A., Sharma D., Sharma R. (2019). Nano-rifabutin entrapment within glucan microparticles enhances protection against intracellular *Mycobacterium tuberculosis*. Artif. Cells Nanomed. Biotechnol..

[211] Kisich K.O., Gelperina S., Higgins M.P., Wilson S., Shipulo E., Oganesyan E., Heifets L. (2007). Encapsulation of moxifloxacin within poly (butyl cyanoacrylate) nanoparticles enhances efficacy against intracellular *Mycobacterium tuberculosis*. Int. J. Pharm..

[212] Faria S., Joao I., Jordao L. (2015). General overview on nontuberculous mycobacteria, biofilms, and human infection. J. Pathog..

[213] Khan O., Chaudary N. (2020). The Use of Amikacin Liposome Inhalation Suspension (Arikayce) in the Treatment of Refractory Nontuberculous Mycobacterial Lung Disease in Adults. Drug Des. Dev. Ther..

[214] Gaspar M.M., Neves S., Portaels O., Pedrosa J., Silva M.T., Euge M. (2000). Therapeutic efficacy of liposomal rifabutin in a *Mycobacterium avium* model of infection. Antimicrob. Agents Chemother..

[215] Blanchard J.D., Valerie Elias A., Cipolla D., Gonda I.L.E.B. (2018). Effective treatment of *Mycobacterium avium subsp. hominissuis* and *Mycobacterium abscessus* species infections in macrophages, biofilm, and mice by using liposomal ciprofloxacin. Antimicrob Agents Chemother..

[216] Golia A., Mahmood B.R., Fundora Y., Thornby K.A., Chahine E.B. (2020). Amikacin liposome inhalation suspension for *Mycobacterium avium* complex lung disease. Sr. Care Pharm..

[217] https://www.fda.gov/news-events/press-announcements/fda-approves-new-antibacterial-drug-treat-serious-lung-disease-using-novel-pathway-spur-innovation.

[218] Shirley M. (2019). Amikacin liposome inhalation suspension: A review in *Mycobacterium avium* complex lung disease. Drugs.

[219] Zhang J., Leifer F., Rose S., Chun D.Y., Thaisz J., Herr T., Nashed M., Joseph J., Perkins W.R., DiPetrillo K. (2018). Amikacin liposome inhalation suspension (ALIS) penetrates non-tuberculous mycobacterial biofilms and enhances amikacin uptake into macrophages. Front. Microbiol..

[220] Rose S.J., Neville M.E., Gupta R., Bermudez L.E. (2014). Delivery of aerosolized liposomal amikacin as a novel approach for the treatment of nontuberculous mycobacteria in an experimental model of pulmonary infection. PLoS ONE.

[221] Griffith D.E., Eagle G., Thomson R., Aksamit T.R., Hasegawa N., Morimoto K., Addrizzo-harris D.J., Donnell A.E.O. (2018). Amikacin liposome inhalation suspension for treatment-refractory lung disease caused by *Mycobacterium avium* complex (CONVERT). Am. J. Respir. Crit. Care Med..

[222] Olivier K.N., Grif D.E., Eagle G., Ii J.P.M., Micioni L., Liu K., Daley C.L., Winthrop K.L., Ruoss S., Addrizzo-harris D.J. (2017). Randomized trial of liposomal amikacin for inhalation in nontuberculous mycobacterial lung disease. Am. J. Respir. Crit. Care Med..

[223] https://clinicaltrials.gov/.

