# Supplementary material for: Nanobiosystems for Antimicrobial Drug-Resistant Infections
Source: Nanomaterials (Basel). 2021 Apr 22;11(5):1075. doi: 10.3390/nano11051075 (PMC8143556; doi:10.3390/nano11051075)
Supplement: Supplementary file 1 [file nanomaterials-11-01075-s001.zip › nanomaterials-1182202-SI.pdf]

Nanobiosystems for antimicrobial drug-resistant infections *By*: F. Gkartziou ; N.s Giormezis; I. Spiliopoulou; S. G. Antimisiaris

Table S1. Clinical Studies involving nanosystems to treat bacterial infections (www.clinicaltrials.gov/ 24-03-2021)

Rank	Title	Status	Results	Conditions	Interventions	URL
**LIPOSOMES**						
**1**	Study to Evaluate ALIS (Amikacin Liposome Inhalation Suspension) in Participants With Nontuberculous Mycobacterial Lung Infection Caused by *Mycobacterium avium* Complex	Recruiting	-	*Mycobacterium* Infections, Nontuberculous	Drug: ALISǀDrug: Azithromycinǀ Drug: Ethambutolǀ Drug: ELC (matching placebo for **ALIS**)	https://ClinicalTrials.gov/show/NCT04677569
**2**	Study to Evaluate Efficacy of LAI When Added to Multi-drug Regimen Compared to Multi-drug Regimen Alone	Completed	+		Drug: **LAI** (Liposomal Amikacin for Inhalation) 590 mg	https://ClinicalTrials.gov/show/NCT02344004
**3**	Liposomal Amikacin for Inhalation (LAI) for Nontuberculous Mycobacteria		+		Drug: Liposomal amikacin for inhalation (**LAI**)ǀDrug: placebo	https://ClinicalTrials.gov/show/NCT01315236
**4**	Safety and Tolerability Study of 2 Dose Level of Arikayce™ in Patients With Bronchiectasis and Chronic Infection Due to *Pseudomonas aeruginosa*.		+	Bronchiectasis	Drug: 280 mg **Arikayce™**ǀDrug: Matching Placebo for Cohort 1ǀDrug: 560 mg Arikayce™ǀDrug: Matching Placebo for Cohort 2	https://ClinicalTrials.gov/show/NCT00775138
**5**	Liposomal Amikacin for Inhalation (LAI) in the Treatment of *Mycobacterium abscessus* Lung Disease		-	*Mycobacterium* Infections, Nontuberculousǀ Atypical	Drug: **LAI** plus multi-drug regimen	https://ClinicalTrials.gov/show/NCT03038178
**6**	Extension Study of Liposomal Amikacin for Inhalation in Cystic Fibrosis (CF) Patients With Chronic *Pseudomonas aeruginosa* (Pa) Infection		+	Cystic Fibrosis	Drug: **Liposomal amikacin** for inhalation	https://ClinicalTrials.gov/show/NCT01316276
**7**	Trial on the Safety of a **New Liposomal Adjuvant System**, CAF01, When Given With the Tuberculosis Subunit Vaccine Ag85B-ESAT-6 as Two Injections With Two Months Interval to Healthy Adult Volunteers		-	Tuberculosis	Biological: 50 μg Ag85B-ESAT-6 aloneǀ Biological: 50 μg Ag85B-ESAT-6 + 125/25 μg CAF01ǀ etc..	https://ClinicalTrials.gov/show/NCT00922363
**8**	Liposomal Amphotericin B for the Treatment of Cryptococcal Meningitis	Unknown	-	Cryptococcal Meningitis	Drug: **Liposomal amphotericin B**ǀDrug: Amphotericin B-deoxycholate	https://ClinicalTrials.gov/show/NCT02136030
**9**	Efficacy of Intrathecal Administration of Liposomal Amphotericin B in Cryptococcal Meningitis		-		Drug: Liposomal amphotericin B	https://ClinicalTrials.gov/show/NCT02686853
**10**	Study to Evaluate Arikayce™ in CF Patients With Chronic *Pseudomonas aeruginosa* Infections	Completed	+	*Pseudomonas aeruginosa* Infection	Drug: Liposomal amikacin for inhalation (**Arikayce™**) using the PARI Investigational eFlow® Nebulizer.ǀ Drug: Tobramycin inhalation solution using a PARI LC® Plus nebulizer.	https://ClinicalTrials.gov/show/NCT01315678
**11**	Study to Demonstrate the Safety of WBR Administered at the Same Time as Intrathecal Liposomal Cytarabine (DepoCyte®) Versus Intrathecal Liposomal Cytarabine (DepoCyte®) Administered After WBR for the Treatment of Solid Tumour Neoplastic Meningitis in Patients With or Without Brain Metastasis.		-	Solid Tumour Neoplastic Meningitisǀ Brain Metastases	Drug: Whole Brain Radio Therapy (WBRT) with sequential Depocyteǀ Drug: Whole brain radiotherapy (WBRT) with concomitant Depocyte	https://ClinicalTrials.gov/show/NCT00854867
**12**	DepoCyt Therapy in Patients With Neoplastic Meningitis From Lymphoma or a Solid Tumor		-	Meningeal Neoplasms	Drug: Intrathecal (injected into the spinal fluid) DepoCytǀ Drug: Intrathecal methotrexateǀ Drug: Intrathecal cytarabine (ara-C)	https://ClinicalTrials.gov/show/NCT00029523
**NANOPARTICLES**						
1	Topical Silver Nanoparticles for Microbial Activity	Recruiting	-	Foot Infection Fungalǀ Infection, Bacterial	Drug: Silver nanoparticles Drug: Topical approved anti-microbial gel	https://ClinicalTrials.gov/show/NCT03752424
